# *Candida albicans* aspartyl protease (Sap6) inhibits neutrophil function via a “Trojan horse” mechanism

**DOI:** 10.1038/s41598-025-91425-x

**Published:** 2025-02-26

**Authors:** Marcin Zawrotniak, Dorota Satala, Magdalena Juszczak, Grażyna Bras, Maria Rapala-Kozik

**Affiliations:** 1 Department of Comparative Biochemistry and Bioanalytics, Faculty of Biochemistry, Biophysics and Biotechnology, Jagiellonian University, Kraków, Poland; 2 Doctoral School of Exact and Natural Sciences, Jagiellonian University, Kraków, Poland

**Keywords:** *Candida albicans*, Neutrophils, Aspartic proteases, Apoptosis, Neutrophil extracellular traps, Reactive oxygen species, Infection, Fungal infection, Fungal host response, Fungal immune evasion

## Abstract

*Candida albicans*, a prevalent fungal pathogen, employs aspartyl proteases such as Sap6 to evade immune defenses, challenging our understanding of host‒pathogen interactions. This research examined the impact of Sap6 on neutrophil responses, which are crucial for innate immunity. Employing flow cytometry and fluorescence microscopy, we explored how Sap6 affects neutrophil functions, particularly by focusing on reactive oxygen species (ROS) production, neutrophil extracellular traps release (NETosis), and apoptosis. Our findings revealed Sap6’s unique ability to bind and internalize in neutrophils, significantly attenuating ROS production through proteolytic damage to NADPH oxidase, resulting in blocking the ROS-dependent NETosis pathway. This disruption in neutrophil functions by Sap6 suggested the presence of a ‘Trojan horse’ mechanism by *C. albicans*. This mechanism reveals a sophisticated immune evasion strategy, shedding light on fungal pathogenicity and host immune interactions. Understanding fungal proteases in immune modulation could inspire new therapeutic approaches for fungal infections.

**Supplementary Information:**

The online version contains supplementary material available at 10.1038/s41598-025-91425-x.

## Introduction

*Candida albicans* is a leading opportunistic fungal pathogen that predominantly affects immunocompromised individuals. These infections are notoriously challenging to treat and often lead to severe health complications and high mortality rates^1^. *C. albicans* pathogenicity is attributed to a complex interplay of various virulence mechanisms, enabling it to colonize host tissues and evade immune responses. Notably, *C. albicans* can switch between noninvasive yeast-like cells and a more virulent filamentous hyphal form. This pathogen also forms biofilms, which are complex multicellular structures that provide a protective environment and facilitate cell communication through quorum sensing molecules (QSMs)^2–5^.

A key component of *C. albicans* virulence is the family of secreted aspartyl proteases (Saps), which consists of at least ten members (Sap1-Sap10), each encoded by distinct genes and exhibiting different expression patterns dependent on the morphological form and environmental conditions^6^. The optimal pH range for Saps activity is between 3 and 5^7,8^. Sap4-Sap6, produced by the filamentous form, play a significant role in tissue colonization^8^ and degrade various host proteins, including those of the epithelial barrier and immune system, facilitating the spread of yeast infection^9,10^. The strong immunomodulatory activity of Sap6 stems from its RGD (RGDRGD) sequence motif (arginine-glycine-aspartic acid), which is critical for proteolytic activity, substrate specificity, and binding capabilities^11–13^. This motif was identified as important for interacting with protease-activated receptors (PARs) and integrins expressed on various cell types^14^, thereby modulating the immune response. Sap6 is both secreted and associated with the surface of the filamentous form of *Candida albicans*, enhancing its ability to interact with host cells^10^. Compared to other members of the Sap family, Sap6 demonstrates a higher level of expression during tissue invasion, particularly in conditions mimicking infection, highlighting its pivotal role in pathogenesis^15^.

Neutrophils are key players in the immune response against *C. albicans* and use various intra- and extracellular mechanisms to counteract early-stage infections. This includes the production of reactive oxygen species (ROS), the release of antimicrobial peptides and proteins, and phagosome acidification. Neutrophils also release neutrophil extracellular traps (NETs) to catch and kill pathogens outside the cell^16,17^. NETs are composed of decondensed chromatin and biocidal granular proteins that immobilize and neutralize pathogens at the infection site^18^. Activation of neutrophil extracellular traps release (NETosis) involves several signaling pathways, with ROS serving as key mediators^19^. The neutrophil response to *C. albicans* includes the recognition of fungal cell wall components, such as glucans and mannans, as well as Saps anchored or released into the extracellular space^3^. However, these defenses can be overwhelmed in advanced infections^16^. Some evidence suggests that phagocytosis and NETosis are most effective in the early infection stages when fewer yeast cells are present. Phagocytosing the larger hyphal form of fungal cells poses additional challenges^3,20,21^. Moreover, increasing Sap concentrations during infection progression has been shown to inhibit NET release^3^.

However, the current understanding of the molecular mechanisms by which *C. albicans* neutralizes the neutrophil antifungal responses is limited. Certain studies suggest that *C. albicans* is capable of surviving within macrophages, a survival strategy potentially aided by the Sap4-6^6^. Neutrophil proteases, such as cathepsin D, and antifungal peptides may directly target fungal proteases within the phagolysosome that are crucial for microbial destruction. Alternatively, Saps might interfere with key metabolic enzymes in macrophages, which are vital for effective microbial elimination^22^. In studies using A549 epithelial cells, both Sap4 and Sap6 were shown to bind to surface integrins, leading to endocytosis and subsequent lysosomal membrane degradation, resulting in cell apoptosis^23^. Additionally, *C. albicans* has evolved strategies to counteract the oxidative responses of immune cells by activating protective enzymes, including catalase and superoxide dismutase, to evade immune detection and destruction^24^. However, the detailed mechanisms through which Saps impair the neutrophil response, particularly in the context of NET production and the overall immune response, have not yet been fully elucidated. This gap in knowledge presents a significant challenge in understanding and effectively combating *C. albicans* infections.

In our study, we aimed to elucidate the role of the aspartyl protease (Sap6) in modulating neutrophil responses against *C. albicans*. We hypothesize that Sap6 employs a ‘Trojan horse’ mechanism, impairing cellular defense functions. Our findings suggest that once internalized by neutrophils, Sap6 can degrade ROS, including NADPH oxidase, thereby inhibiting NET production and inducing apoptotic death. This mechanism represents another defensive strategy of *C. albicans* against the immune system.

## Results

### **Sap6 internalization by neutrophils leads to enzyme accumulation and resulting in apoptotic cell death**

**Fig. 1:**
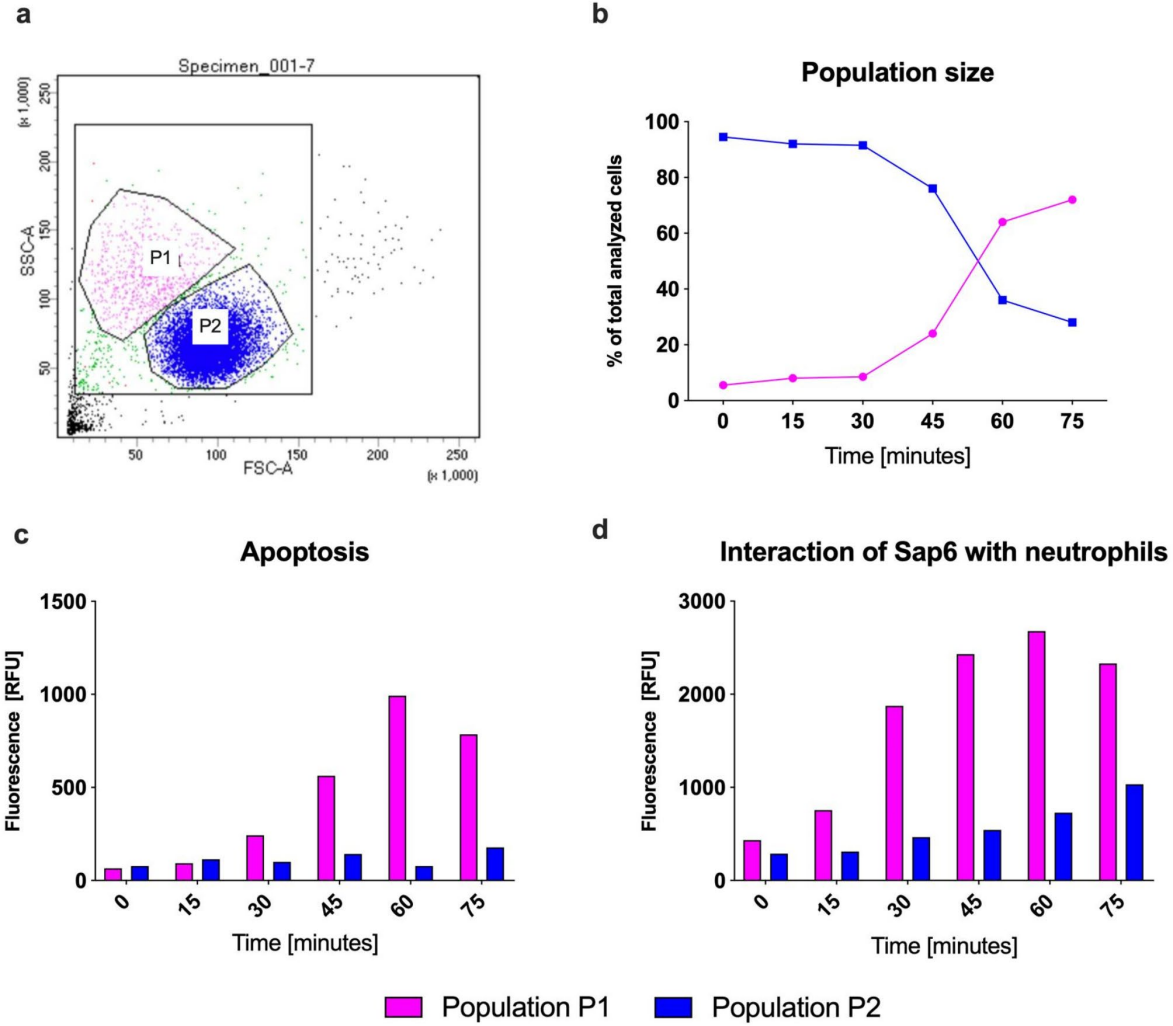
Analysis of the interaction of Sap6 with neutrophils, cell apoptosis, and NETosis at the selected time points. Neutrophils (1 × 10^6^ cells/sample) were incubated with fluorescently labeled Sap6 at a concentration of 100 ng/ml for up to 75 min. The cells were fixed at 15-minute intervals and Flow cytometry was used for their analysis. (a) Morphological characteristics identifying two distinct cell populations observed after 30 min of incubation; (b) variation in the proportion of each population over time, expressed as a percentage of all cells analyzed; (c) temporal analysis of apoptosis using CellEventTM Caspase-3/7 Green Detection Reagent and (d); temporal binding patterns of Sap6 to the neutrophil populations. This experiment was repeated three times, and the results are representative of these replicates.

Neutrophils treated with Sap6 at a concentration of 100 ng/ml were subjected to cytometric analysis at subsequent time intervals. Sap6 concentrations were chosen based on analyses of responses to different concentrations, as outlined later in the manuscript. This analysis revealed two distinct, morphologically differentiated populations of neutrophils (P1 and P2) based on forward scatter (FSC) and side scatter (SSC) characteristics, as illustrated in Fig. 1A. The sizes and proportions of these populations were observed to change over time (Fig. 1B). Approximately 30 min after exposure to Sap6, an increase in the size of the P1 population was noted, while the size of the P2 population decreased. The neutrophils in the P2 population appeared to represent native-state cells. This shift is associated with the activation of proapoptotic caspases, as indicated by an increase in the fluorescence intensity of the CellEvent Caspase 3/7 Detection Reagent. (Fig. 1C).

Using fluorescently labeled Sap6, its direct interaction with neutrophils was confirmed. Cytometric analysis revealed that beginning at the onset of contact between the protease and the neutrophils, the average fluorescence intensity per cell increased in proportion to the amount of protease present. This enhancement suggested that the enzyme directly interacted with the neutrophils (Fig. 1D). However, these interactions varied between the two analyzed cell populations. In apoptotic cells (P1), the fluorescence level increased fivefold within the first hour of contact. In contrast, in the P2 population, the increase in fluorescence intensity was more gradual, doubling the initial level after one hour.

Microscopic analysis corroborated the cellular response to Sap6. A considerable number of neutrophils maintain the characteristic of segmented nuclear shape and exhibit increased proapoptotic caspase activity (Supplementary, Fig. 1). Notably, these cells showed significant internal accumulation of the Sap6. Time-lapse analysis revealed an initial buildup and increase in the concentration of the Sap6 in neutrophils, followed by apoptosis activation (Supplementary, Movie 1). The localization of Sap6 within neutrophils seems to be a critical factor in determining the cellular response mechanism to this yeast protein.

**Fig. 2:**
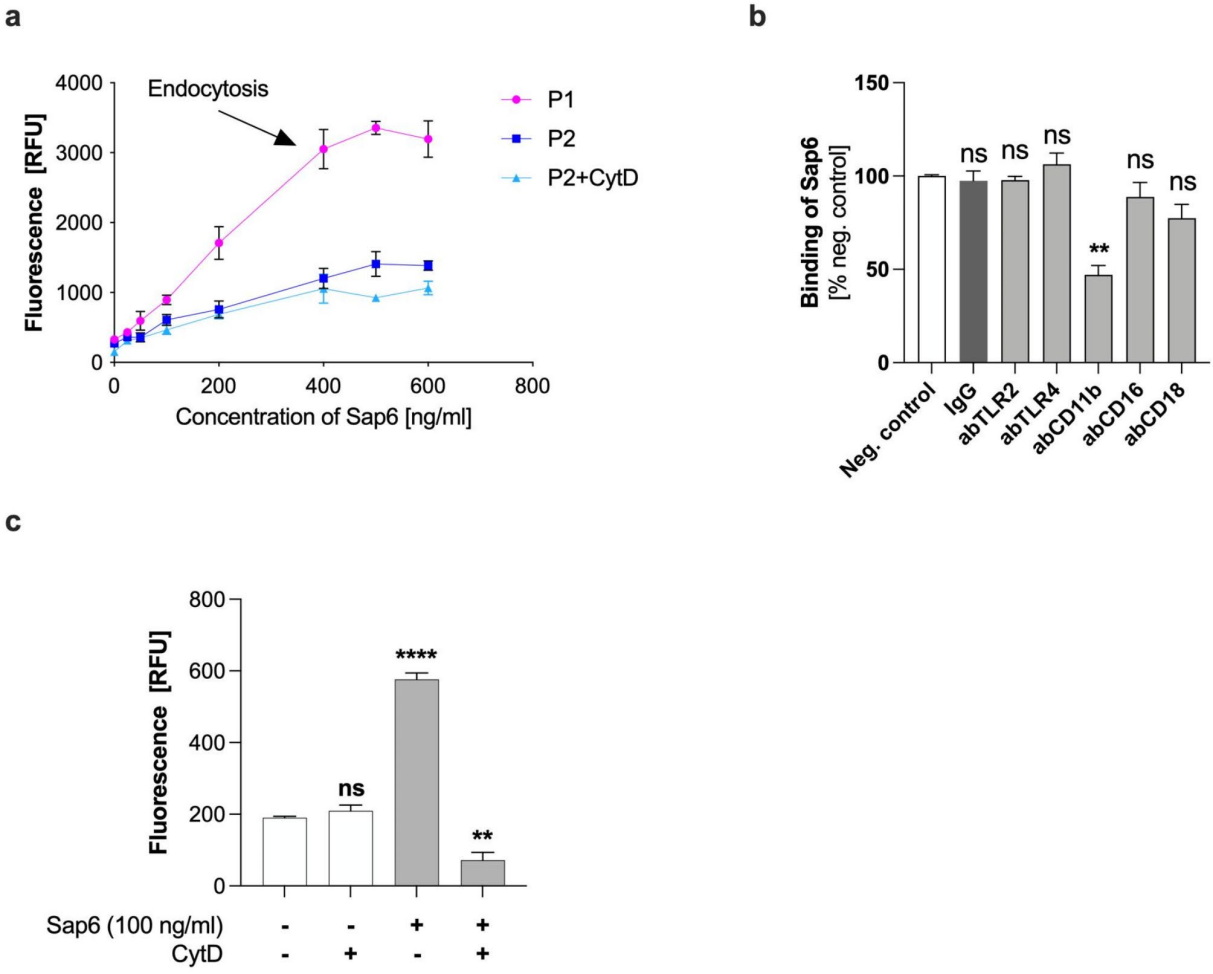
Sap6 binding to neutrophils with active or inhibited phagocytosis. (a) Native or CytD-treated neutrophils for phagocytosis inhibition were incubated for 1 h with fluorescently labeled Sap6 at various concentrations (0-600 ng/ml) and subjected to flow cytometric analysis. The presented graphs depict the fluorescence intensity of neutrophils in the Sap6 fluorescence channel for population P1 and P2. (b) Neutrophils were preincubated for 30 min with antibodies targeting TLR2, TLR4, CD11b, CD16, and CD18, incubated with fluorescently labeled Sap6 (100 ng/ml) for 1 h and subjected to flow cytometric analysis (all populations). The graph depicts Sap6 binding to neutrophils following receptor blockade, expressed as a percentage relative to the untreated control group. The data are presented as the means ± SEMs from four independent experiments. Asterisks indicate statistically significant differences compared to untreated cells (**p < 0.01; ns, no significant difference). (c) Neutrophils were stained with CellEvent™ Caspase-3/7 Green Detection Reagent and then treated to either maintain active endocytosis (– CytD) or inhibit it (+ CytD). These cells were then incubated with 100 ng/ml Sap6 for 1 h, followed by fixation and analysis using flow cytometry. The graph shows the mean fluorescence intensity in the green channel, indicative of caspase 3 and 7 activation. The data represent the mean ± SEM of three independent experiments. The difference between untreated cells and treated samples is indicated by asterisks (**p < 0.01, ****p < 0.001; ns - not significant).

**Fig. 3:**
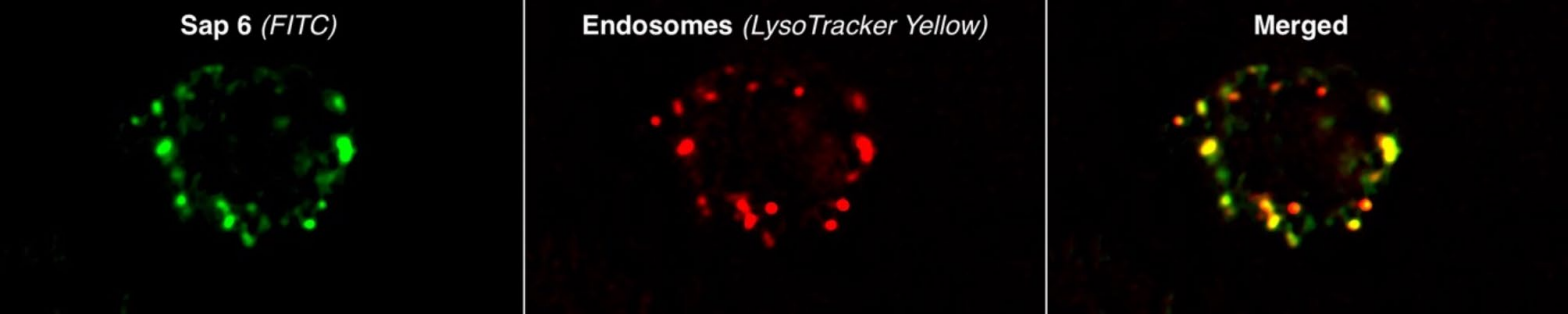
Colocalization of Sap6 with acidic endosomes. Neutrophils were preincubated with LysoTracker Yellow for 15 min to label acidic endosomes. Following washing, the cells were incubated with FITC-labeled Sap6 for 1 h. After subsequent washing and fixation, the cells were examined using fluorescence microscopy to assess the colocalization of Sap6 with acidic endosomes.

From the host’s perspective, endocytosis of Sap6 aims to neutralize foreign molecules from the extracellular space. However, the internal accumulation of this protease within neutrophils alters their cellular response. We employed flow cytometric analysis to assess the interaction between Sap6 and neutrophils. Cells with active and inhibited endocytosis by cytochalasin D (5 µM) were incubated with various concentrations of fluorescently labeled protease for 60 min, after which the fluorescence levels were analyzed. Apoptotic cells (population P1) exhibited an approximately threefold greater level of protease binding (Fig. 2A; P1) than native cells (population P2) (Fig. 2A; P2). Interestingly, inhibition of the endocytosis mechanism in neutrophils significantly reduced the size of the P1 population (Supplementary. Figure 3), demonstrating that the morphological change in cells and the shift between populations is related to active protease endocytosis. These findings suggested that Sap6 not only adheres to the neutrophil surface but is also actively internalized and sequestered within these immune cells. Within the P2 population, no significant difference in the extent to which Sap6 bound to neutrophils via either active or inhibited endocytosis was observed. These findings suggested that in the P2 population, Sap6 primarily binds to the cell surface without being internalized.

To ascertain which neutrophil surface receptor is pivotal for Sap6 endocytosis, we conducted cytometric analyses of the interaction between fluorescently labeled Sap6 (100 ng/ml) and neutrophils in the presence of antibodies that block selected receptors. The binding of Sap6 to these surface receptors is a critical step preceding its endocytosis.

To identify the implicated receptor, antibodies targeting five different types of receptors, TLR2, TLR4, CD11b, CD16, and CD18, were used. These findings suggest that the CD11b receptor plays a key role in recognizing of Sap6 (Fig. 2B). A decrease in fluorescence intensity in the cells with the blocked CD11b receptor indicated a reduced interaction between Sap6 and this surface protein, consequently impeding the endocytosis of Sap6.

To determine whether Sap6 accumulation in immune cells is essential for triggering cell death mechanism, neutrophils were preincubated with 5 µM cytochalasin D, before being exposed to 100 ng/ml Sap6. Subsequently, the activity of caspase 3/7 in these cells was assessed using fluorescence methods (Fig. 2C). We can conclude that endocytosis inhibition prevents the activation of proapoptotic caspases in the presence of Sap6. These findings underscore the importance of Sap6 internalization and accumulation within neutrophils as a crucial step in initiating apoptosis.

The cytometric study was supported by detailed microscopic analysis, where we utilized fluorescently labeled Sap6 (Alexa Fluor 555) to observe protein internalization by neutrophils in real time. The cells were placed in a 100 ng/ml proteinase solution, and microscopic observations were conducted over a period of 45 min, with the protein remaining in the solution.

The results (Supplementary, Movie 2) demonstrated that over the duration of contact, Sap6 accumulated on the cell surface and within the neutrophils (indicated by the red dots that spread throughout the cell). Notably, after approximately 30 min, there was a significant increase in the fluorescence intensity in the red channel.

Given that the fluorescence intensity correlates linearly with fluorophore concentration, it can be inferred that the intracellular concentration of Sap6 increased by 4–9 times compared to its concentration in solution (with the average background signal being approximately 200 RFU and the intensity of the spots within neutrophils ranging from approximately 800 to 2000 RFU). Considering the molecular weight of the protease (approximately 40 kDa), transmembrane transport and internalization are likely achieved through active endocytosis, leading to the accumulation of Sap6 inside the neutrophils.

### Sap6 localizes to endosomes and damages the mechanism of reactive oxygen species generation in neutrophils

Knowing that Sap6 accumulates within neutrophils, we identified compartments where this protease could be localized. For this purpose, neutrophils were preincubated with the endosome marker LysoTracker Yellow, and then, the cells were incubated with 100 ng/ml fluorescein isothiocyanate (FITC)-labeled Sap6 for 1 h at 37 °C and imaged microscopically after fixation. The partial spatial colocalization of LysoTracker (red) and Sap6 (green) fluorescence signals indicated areas where Sap6 was in an acidic environment (Fig. 3). The results showed that once internalized, the yeast protease is exclusively stored in endosomes. However, there are also regions where colocalization did not occur, suggesting a possible ongoing fusion process. This process involves the merging of neutral endosomal vesicles containing Sap6 with acidic lysosomes^25,26^. Notably, Sap6 within neutrophil endosomes does not lead to lysis, which could trigger apoptotic mechanisms, as observed in A549 cells^23^.

.

**Fig. 4:**
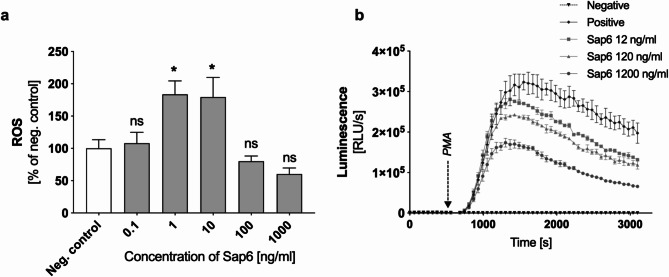
Release of ROS by neutrophils in response to Sap6 treatment. (a) Each sample, containing 2 × 10^5^ neutrophils, was labeled with rhodamine-123 and subsequently incubated with Sap6 at concentrations ranging from 0.1 to 1000 ng/ml for 30 min. Control cells were not treated with Sap6. After washing, the neutrophils were analyzed for fluorescence levels using flow cytometry. The data are presented as the means ± SEMs from three independent replicates. Asterisks indicate statistically significant differences compared to untreated cells (*p < 0.05; ns - not significant). (b) Effect of Sap6 on ROS release in response to PMA. The influence of Sap6 on neutrophil ROS production was evaluated using a chemiluminescence assay. Neutrophils (1 × 10^6^/sample) were incubated with Sap6 at concentrations of 12, 120, and 1200 ng/ml for 1 h, followed by suspension in a luminol solution. PMA (25 nM) was added 10 min later, and the luminescence level was monitored at 60-second intervals. The results are presented as luminescence kinetics over time. The data are presented as the means ± SEMs from three independent replicates.

Furthermore, ROS production in neutrophils exposed to varying concentrations of Sap6 was investigated (Fig. 4A). For this purpose, cells were first incubated with rhodamine-123 and then treated with different concentrations of Sap6, after which the fluorescence intensity was measured after 1 h to determine the ROS levels. An observed twofold increase in intracellular ROS was noted at Sap6 concentrations of 1–10 ng/ml. In this range, Sap6 activates ROS-dependent NETosis pathway^3^. However, at higher Sap6 concentrations, between 100 ng/ml and 1000 ng/ml, the ROS production was at level of control cells. Given that, Sap6 concentrations above 10 ng/ml could indicate an inhibitory effect of Sap6 on intracellular ROS generation mechanisms.

To assess the impact of Sap6 on neutrophil oxidative mechanisms, chemical induction of reactive oxygen species (ROS) was performed using phorbol ester (PMA) to stimulate protein kinase C (PKC), which leads to the rapid release of ROS. This assessment utilized a luminescent, luminol-based technique. Neutrophils were first incubated with Sap6 at selected concentrations for 15 min. ROS production was then activated by the addition of 25 nM PMA, and luminescence measurements were conducted in the presence of luminol to analyze changes in ROS levels. The results (Fig. 4B) indicated the marked influence of Sap6 on the amount of ROS released by neutrophils. A notable decrease in ROS release was observed with increasing Sap6 concentration, particularly within the range of 120–1200 ng/ml, where up to a 50% reduction in ROS levels compared to those in the control was noted.

Importantly, while Sap6 did not alter the initiation of ROS release, it did affect the efficiency of this process. The observed inhibitory effect of Sap6 on ROS production might be attributable to the protease’s influence on both the signaling pathway that activates ROS generation and the direct effect on the ROS-producing NADPH oxidase.

### Modulation of ROS production by Sap6 results from its proteolytic activity inside the cell

**Fig. 5:**
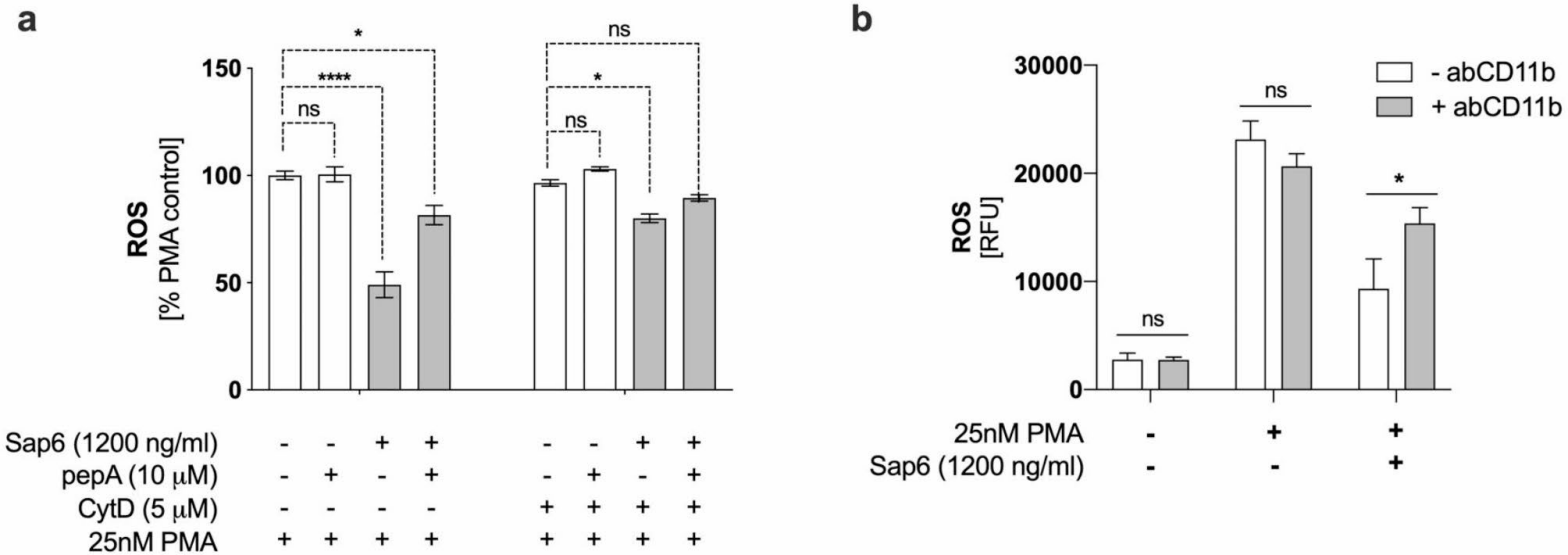
Effects of proteolytic activity and endocytosis on ROS production in the presence of Sap6. (a) Neutrophils (1 × 10^6^) with either active or inhibited (+ CytD) endocytosis were exposed to Sap6 at a concentration of 1200 ng/ml, along with a protease inhibitor (pepA), for 1 h. This was followed by the induction of ROS production using phorbol esters (PMA). The results present the total luminescence observed at 1 h. The data are expressed as the means ± SEMs from three independent replicates. (b) PMN cells were incubated with anti-CD11b (Mac-1) receptor antibodies to block the receptor. As a control, neutrophils with an unblocked Mac-1 receptor were used. These cells were then exposed to Sap6 at a concentration of 1200 ng/ml, followed by activation with PMA. The data shown represent the mean total luminescence ± SEM from three replicates. Asterisks indicate statistically significant differences between the positive control and the samples (**p < 0.01, ****p < 0.001, ns - not significant).

To determine how Sap6 affects neutrophils, specifically by impairing their ROS generation system, we explored both the endocytosis and proteolytic activity of this yeast enzyme. Neutrophils preloaded with rhodamine-123 were treated with 5 µM cytochalasin D for 15 min to inhibit endocytosis. Subsequently, these cells were incubated for 15 min with either active or inhibited Sap6 at a concentration of 1200 ng/ml—the concentration at which the most pronounced effect on ROS inhibition occurred. ROS release was then chemically activated using 25 nM PMA. Cytometric analysis after 1 h revealed that ROS production in neutrophils with hindered endocytosis in the presence of Sap6 was significantly greater (approximately 80%) than that in cells capable of internalizing the protease (approximately 50%) (Fig. 5A). These findings suggest that internalization and accumulation within neutrophils are key steps leading to impaired ROS release. The extracellular presence of the protease did not significantly affect the PMA-induced response.

Inhibition of the proteolytic activity of Sap6 has also been proven to be crucial for suppressing ROS production. Compared with those treated with the active protease, neutrophils treated with the pepstatin A-inhibited Sap6 exhibited more efficient ROS production (approximately 80%). This effect occurred partly because pepstatin A has low inhibitory activity against Sap6 (IC50 > > 1.2 mM), but higher concentrations of pepstatin A could negatively impact neutrophils^27^. Additionally, simultaneously blocking endocytosis and proteolytic activity of the enzyme demonstrated that the ROS production levels matched those of the control cells treated with PMA. These findings support the hypothesis that ROS production is regulated by both blockade of endocytosis and effective inhibition of enzyme activity. These results unequivocally indicate that the reduction in ROS production in neutrophils in contact with Sap6 is dependent on the intracellular proteolytic activity of the enzyme.

We further investigated whether blocking Mac-1 (anti-CD11b antibody), which leads to impaired uptake of Sap6 from the extracellular environment, affects ROS production in neutrophils in the presence of the Sap6. To this end, rhodamine-123-labeled neutrophils with antibody-mediated blockade of the CD11b receptor were incubated with Sap6 at a concentration of 1200 ng/ml. Subsequently, ROS production was chemically induced, and the cells were analyzed via flow cytometry after 1 h of PMA treatment. Blocking the CD11b receptor with antibodies alone does not influence ROS production activation by PMA in the absence of Sap6, as observed in control samples. Compared with PMA-stimulated control neutrophils, neutrophils with a blocked Mac-1 receptor in the presence of the active Sap6 responded to PMA stimulation by producing ROS at a level of 85% compared to PMA-stimulated control neutrophils (Fig. 5B). The 15% decrease in ROS production could be attributed to incomplete blockade of the CD11b receptor by antibodies. Conversely, when the CD11b receptor is not blocked and neutrophils are incubated with Sap6, ROS production is reduced to 50% compared to that in control neutrophils. These findings suggest a critical role for the CD11b receptor in modulating the mechanism of ROS production. Integrin seems to function as a ‘protease gate’, facilitating the intracellular entry and subsequent proteolytic activity of Sap6.

### Sap6 damages oxidative mechanisms in neutrophils through proteolytic degradation of NADPH oxidase

By analyzing the signaling pathways involved in the neutrophil response to PMA, three major mediators—PKC, ERK1/2, and NADPH oxidase—have been identified as potential targets of Sap6. The proteolytic degradation of these proteins by Sap6 could significantly influence ROS production outcomes. To determine which of these proteins could serve as targets for the protease, we specifically investigated the potential effects of Sap6 on each selected protein.

#### PKC

**Fig. 6:**
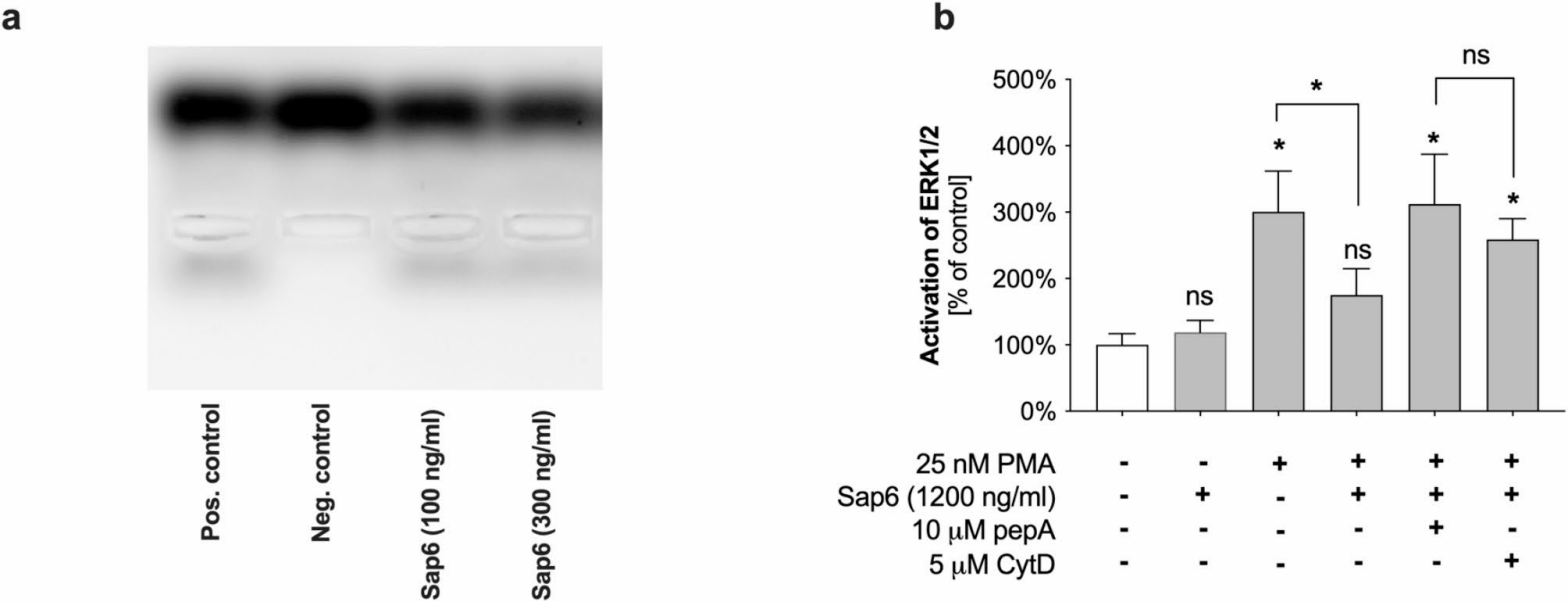
Effects of Sap6 on protein kinase C (PKC) activity and ERK 1/2 phosphorylation. (a) PKC was incubated with Sap6 at concentrations of 100 ng/ml and 300 ng/ml. Next, a protease inhibitor (pepA) was added. The reaction mixture, containing the kinase substrate following the manufacturer’s instructions, was subsequently added to the prepared samples. A sample of PKC without Sap6 served as a positive control, whereas a sample devoid of kinase served as a negative control. After incubation, the samples were subjected to electrophoretic separation. This experiment was repeated three times, and the results are representative of these replicates. (b) To assess the impact of the Sap6 on the ERK1/2 mediator in neutrophils via either active or inhibited endocytosis, the cells were incubated with 1200 ng/ml Sap6. PepA-Sap6 was utilized as a control. Following activation with phorbol esters (PMA), the cells were lysed, and the level of phosphorylated ERK1/2 (p-ERK1/2) was analyzed via ELISA. The data are presented as the means ± SEMs from three independent replicates. Asterisks indicate statistically significant differences between the positive control and the samples (*p < 0.05, ns —not significant).

In an in vitro system, protein kinase C (PKC) activity can be assayed with a synthetic fluorescent peptide (C1) as a substrate. The phosphorylation of this peptide results in a change in charge, which is detectable as a shift in electrophoretic mobility during gel electrophoresis. PKC kinase was incubated with the Sap6 at concentrations of 100 ng/ml and 300 ng/ml in a pH 5.5 environment, mimicking endosomal conditions. Subsequently, the activity of PKC kinase was assessed. The findings are represented by photographic documentation of gel electrophoretic separation and accompanying quantitative densitometric analysis (Fig. 6A). The presence of a peptide band below the sample application site on the gel indicated the product of PKC activity, which was the focus of the densitometric analysis. Lane 1 on the gel represents a positive control, showing active PKC incubated with the C1 peptide. Lane 2 is a negative control and displays the location of the peptide alone. Lanes 3 and 4 show the products of PKC activity in which PKC was incubated with the Sap6 at 100 ng/ml and 300 ng/ml, respectively. To prevent potential degradation of the C1 peptide by Sap6, the C1 substrate was added to the solution after the addition of a protease inhibitor (pepstatin A).

The results indicated that the yeast protease did not impair the function of protein kinase C within the studied concentration range of Sap6, as evidenced by the lack of significant differences between the samples in lanes 3 and 4 and the positive control. Therefore, it can be concluded that the observed decrease in ROS levels in PMA-activated neutrophils was not the result of damage to PKC, the first signaling mediator.

#### ERK1/2

The ERK1/2 kinase plays a role in activating NETosis and regulates proapoptotic signaling pathways^28^. Its cascade activation occurs, among other triggers, following the chemical stimulation of neutrophils with PMA. We analyzed ERK1/2 phosphorylation in neutrophils treated with 1200 ng/ml Sap6 using an ELISA. The results indicated that in cells capable of internalizing proteolytically active Sap6, no activation of this kinase was observed post-PMA stimulation (Fig. 6B). When an endocytosis inhibitor was applied, active Sap6 did not significantly reduce ERK1/2 activity. Similarly, inhibition of the Sap6 enabled mediator activation following chemical induction of the NETosis pathway with PMA. These findings suggested that the intracellular presence of the active Sap6 leads to the inhibition of ERK1/2. However, considering that ERK1/2 can both stimulate ROS production and be activated by ROS release, the observed effect could be either a primary action of the Sap6 or a secondary consequence of impairment of the ROS generation mechanism. This uncertainty makes it challenging to conclusively determine whether the effect observed is a direct result of the proteolytic activity of Sap6 or an indirect outcome of disrupted ROS production.

#### NADPH oxidase

NADPH oxidase is a protein complex that assembles and is activated in response to intracellular and extracellular signals. It is believed that the presence of oxidase within endosomes facilitates and accelerates the destruction of foreign molecules^29^. The direct interaction of oxidase subunits with Sap6, especially when they accumulate at high concentrations in endosomes, may lead to proteolytic degradation of these protein complexes. To investigate the potential of the Sap6 to degrade the NADPH oxidase system, we examined the degree of fragmentation of two critical oxidase subunits, gp91^phox^ and p67^phox^, both of which are essential for the functional ROS generation system^30^. The p67^phox^ subunit homolog is a key component of the NADPH oxidase complex that translocates to the membrane of endosomes upon activation^29,31^. It contains an internal activation domain that regulates electron transfer from NADPH to the flavin domain of gp91phox. The gp91^phox^ subunit is the primary catalytic component of NADPH oxidase^32^.

We performed an analysis of potential damage to these subunits via Western blot analysis of lysates from neutrophils treated with the Sap6, both with and without the inhibitor pepstatin A.

**Fig. 7:**
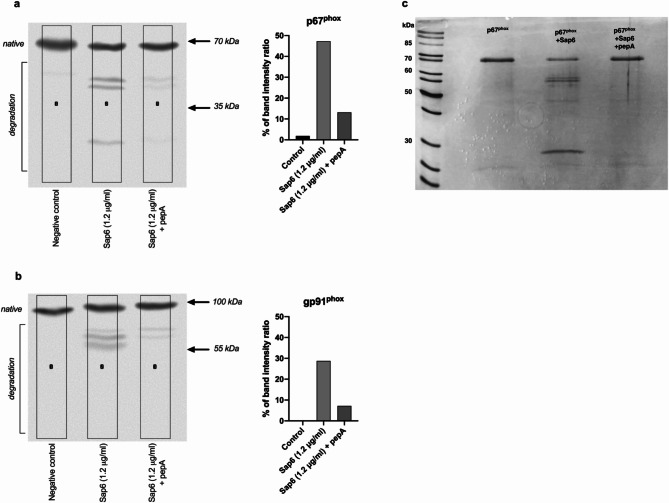
Degradation of NADPH oxidase subunits. To determine the ability of Sap6 to degrade selected subunits of NADPH oxidase, neutrophils (1 × 10^6^ cells/sample) were incubated with Sap6 at a concentration of 1200 ng/ml for 1 h and then lysed. The lysates were subjected to electrophoretic separation and transferred to a membrane, after which the (a) p67^phox^ and (b) gp91^phox^ subunits were identified using antibodies. The graphs show the intensities of the bands corresponding to the degradation products of the individual oxidase subunits. (c) To evaluate the capacity of Sap6 to degrade the p67^phox^ subunit, an experiment was conducted with the isolated protein. The oxidase subunit was incubated with Sap6 at a substrate-to-enzyme ratio of 50:1 for 3 h. This was followed by electrophoretic separation and subsequent staining to visualize the degradation products. As a control to gauge the proteolytic activity of the enzyme, a sample was incubated with a protease inhibitor (pepA) for 30 min prior to the addition of Sap6.

The protein degradation profile analysis revealed the proteolytic degradation of the p67^phox^ subunit by Sap6 in neutrophils (Fig. 7A), identifying fragments with lower masses. The gp91^phox^ subunit (Fig. 7B) also underwent proteolytic degradation, resulting in three fragments of lower masses. In both cases, the use of the Sap6 inhibitor pepstatin A reduced the intensity of the bands, indicating decreased degradation product formation. Densitometric analysis indicated that inhibitor application led to a fourfold decrease in the formation of degradation products. This substantial reduction confirmed that both oxidase subunits can be a subject to degradation by the Sap6 inside neutrophils. The minimal degradation observed in the presence of the inhibitor was attributed to incomplete inhibition of the protease, consistent with the findings from the ROS analysis.

The capacity of Sap6 to degrade NADPH oxidase subunits was further validated in vitro using a commercially available p67^phox^ subunit. The experiment involved incubating Sap6 with the substrate at a 1:50 ratio for 3 h, followed by electrophoretic separation under denaturing conditions (Fig. 7C). The interaction between the p67^phox^ subunit and the Sap6 resulted in extensive fragmentation of the protein. The electrophoretic degradation pattern observed mirrors the results obtained under intracellular conditions, corroborating that Sap6 can cause proteolytic damage to the NADPH oxidase complex.

Such damage can destabilize the NADPH oxidase complex, preventing the release of ROS into endosomal vesicles. This result demonstrated that Sap6 can cause damage to one of the key subunits involved in the formation of an oxidase complex.

### Damage to NADPH oxidase leads to blockage of the NETosis mechanism

**Fig. 8:**
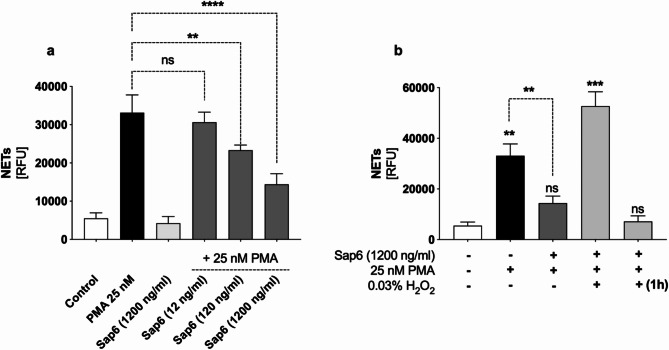
Effect of Sap6 on NETosis. (a) Neutrophils exposed to varying concentrations of Sap6 (12-1200 ng/ml) were activated with PMA, and after 3 h, the extent of NET release was analyzed via fluorescence. The control group consisted of neutrophils not stimulated by PMA. (b) The capacity of NETs to be activated via an extracellular ROS source was also examined. For this purpose, cells were treated with Sap6 and then stimulated with PMA or PMA combined with hydrogen peroxide. Additionally, in one sample, the cells were stimulated with PMA, and hydrogen peroxide was added after 1 h. Similarly, the level of NET release in these samples was assessed through fluorescence analysis. The data are expressed as the means ± SEMs from three independent replicates. Asterisks indicate statistically significant differences between the positive control and the samples (**p < 0.01, ***p < 0.005, ****p < 0.001, ns—not significant).

Contact between neutrophils and low concentrations of Sap6 induces the release of NETs; however, increased concentrations of the Sap6 in the extracellular space inhibit the mechanism of NETosis^3^. An experiment was conducted to determine whether intracellular Sap6 blocks NET release by impairing the ROS production system. Neutrophils were incubated with Sap6 at various concentrations for 30 min, followed by NETosis activation using 25 nM PMA. PMA directly interacts with the intracellular NETosis mediator PKC, leadind to the activation of the classical ROS-dependent NETosis pathway, which is independent of neutrophil surface receptors^33^. For accurate detection of NETs, after 3 h of activation, NETs released into the extracellular space were fragmented using micrococcal nuclease (MNase) as previously described and labeled with SYTOX Green for quantitative fluorescence analysis^3^. With increasing Sap6 concentration, the amount of extracellular DNA decreased (Fig. 8A), which confirmed the effect of Sap6 on ROS production.

To rule out damage to other NETosis signaling mediators, an experiment was conducted with exogenous ROS delivery via hydrogen peroxide treatment. This approach restores NET release in neutrophils via a compromised ROS generation system, as observed in chronic granulomatous disease (CGD) patients^34,35^. Here, neutrophils preincubated with 1200 ng/ml Sap6 were activated for NETosis with 25 nM PMA. One sample was supplemented with 0.03% H_2_O_2_ simultaneously, while the other was supplemented one hour post-PMA treatment. The level of released NETs was quantified as previously described.

Our findings revealed that introducing 0.03% hydrogen peroxide from an extracellular source at the time of neutrophil activation restored NET release capacity (Fig. 8B). These findings suggested that Sap6 accumulation within neutrophils does not damage other crucial components of the NETosis signaling pathway but rather specifically targets the degradation of the intracellular ROS source NADPH oxidase. However, H_2_O_2_ supplementation 1 h after PMA addition did not trigger NET release, indicating that approximately 60 min after Sap6 contact, the ROS production system was blocked, inactivating the NETosis pathway and potentially triggering proapoptotic processes^36^.

Given that the primary NETosis signaling pathway utilizes ROS as a signal mediator, our results demonstrated that damage to oxidative mechanisms due to the intracellular proteolytic activity of Sap6 leads to inhibition of the NETosis response and consequently activates apoptotic cell death mechanisms.

### Sap6 reduces the antimicrobial response efficacy of neutrophils against *Candida albicans*

Blocking NADPH oxidase activity, and consequently ROS-dependent bactericidal mechanisms, can affect neutrophils’ ability to eliminate yeast cellsTo verify this, neutrophils were preincubated with 120 ng/ml Sap6 for 30 min, then co-incubated with *C. albicans* at an MOI (Multiplicity of Infection) of 1 in RPMI-1640. The control consisted of neutrophils not exposed to Sap6. The incubation was carried out for 19 h at 37 °C, with yeast growth analyzed hourly by measuring optical density at 600 nm. To provide further insights into the dose-dependent effects of Sap6, additional results showing the impact of various Sap6 concentrations on *C. albicans* growth have been included in the supplementary data (Supplementary, Fig. 5).

**Fig. 9:**
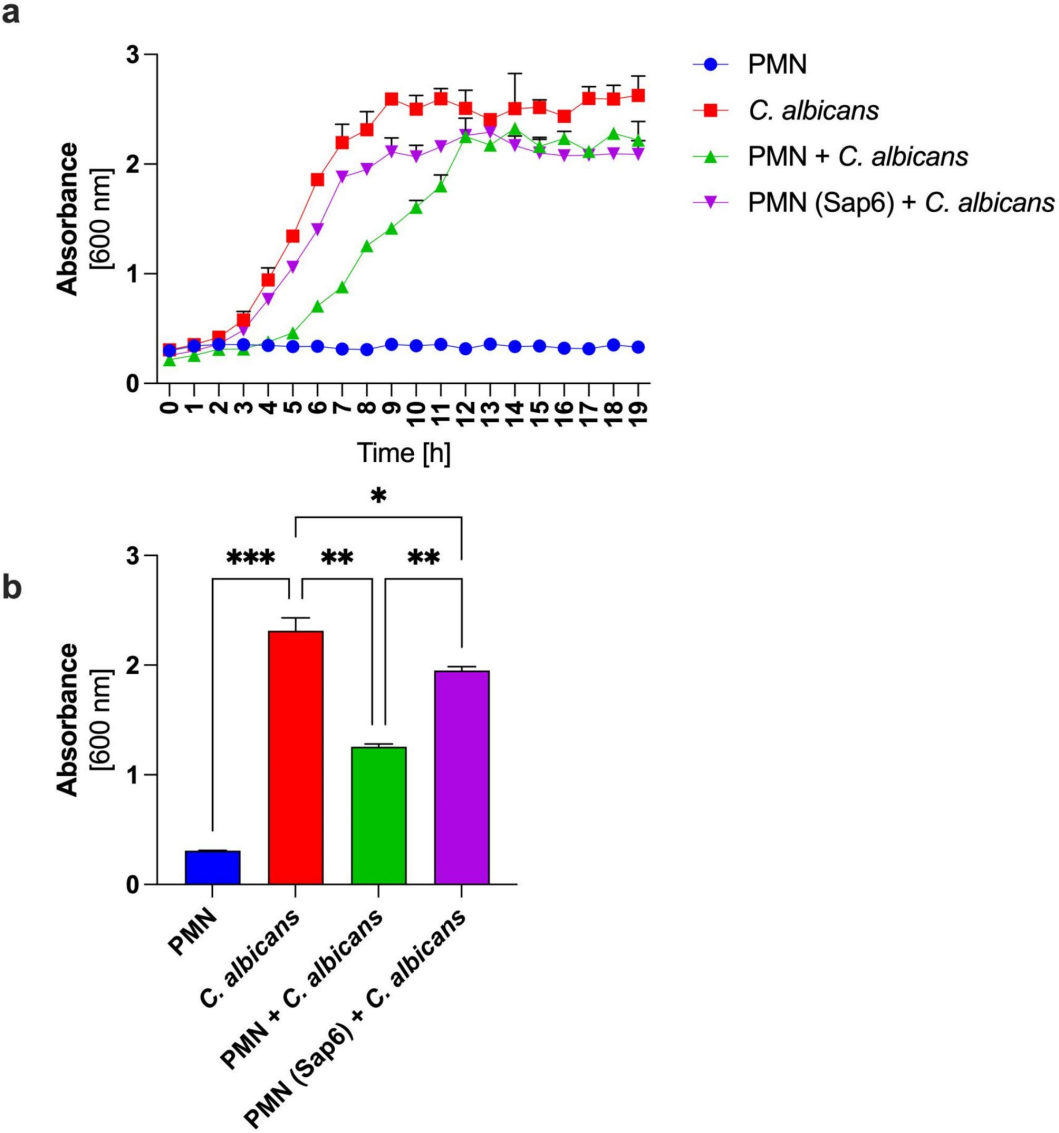
Growth of *C. albicans* in contact with neutrophils. Neutrophils were preincubated with 120 ng/ml Sap6 for 30 min, then co-incubated with C. albicans at an MOI of 1 in RPMI-1640 for 19 h at 37 °C. (a) Yeast growth was analyzed hourly by measuring optical density at 600 nm, (b) the OD values after 8 h of incubation. The negative control consisted of *C. albicans* cells alone, while the positive control consisted of yeast cells co-incubated with neutrophils that were not preincubated with Sap6. The data are expressed as the means ± SEMs from three independent replicates.

The results indicate that the rapid growth of *C. albicans* begins after 3 h of incubation, associated with dynamic morphological changes (Supplementary, Fig. 4), including hyphal development and later biofilm formation (Fig. 9a). The presence of neutrophils delays both the onset of yeast growth (by approximately 2 h) and the growth rate (by approximately 6 h) (Fig. 9b). This effect is linked to antimicrobial mechanisms, reducing the initial number of yeast cells capable of growth. After 5 h of incubation, most neutrophils in contact with the yeast activate antifungal mechanisms, which ultimately lead to neutrophil death and allow the remaining C. albicans cells to proliferate. However, this growth inhibition effect is not observed in neutrophils preincubated with Sap6. This confirms that the shutdown of bactericidal mechanisms and the activation of apoptosis in these cells due to Sap6 internalization significantly impact pathogen development. The presence of the Sap6 in the extracellular space influences the infection’s progression and rate, effectively protecting the yeast from neutrophil mechanisms.

## Discussion

Infections with pathogenic microorganisms typically elicit a swift immune response at the infection site. Neutrophils, pivotal in this process, possess multiple mechanisms to attack and damage invading cells. However, the exact criteria guiding the choice of a specific response mechanism by neutrophils remain unclear. In certain situations, the capabilities of neutrophils are compromised, resulting in early cell apoptosis^37^. One factor contributing to neutrophil apoptosis is the presence of substances released by microorganisms, which serve as a defensive barrier against the host immune system.

Phagocytosis is the primary method employed by neutrophils to eradicate *C. albicans*. This mechanism is notably effective against yeast in the early stages of infection when the number of pathogen cells is limited and when the size of the cells is relatively small^20^. However, *C. albicans* can morphologically transform during infection, and its increased cell size due to filamentation makes phagocytosis less effective or even impossible^38,39^. Studies have shown that the presence of *C. albicans*, particularly in its filamentous form, triggers the release of NETs^3,16^. Nevertheless, the conditions for effective NETosis are stringent, and in the case of a developed fungal biofilm, neutrophils lose their NETosis capability and any effective host defense^20,40^. One agent from yeast that induces NETosis is the Saps, especially Sap6, but the enzyme concentration range that triggers NETosis is quite narrow. Low concentrations (up to 10 ng/ml) are enough to initiate NET release; however, the lack of response to higher concentrations was previously unclear. The findings presented in this article suggest that high concentrations of Sap6 (above 100 ng/ml) can modulate the neutrophil response, effectively negating response to yeast infection.

The exact physiological concentration of Sap6 during infection remains challenging to assess, as it depends on various factors, including the infection stage, *C. albicans* strain, fungal morphological form, and infection site. Furthermore, whether Sap6 is surface-associated or secreted extracellularly significantly influences its local concentration, as surface-bound Sap6 can create high local protease levels. Experimental concentrations in this study were optimized to achieve a clear biological effect on neutrophils and are consistent with previously reported values ranging from 236 to 1075 ng/ml^23^. Importantly, in vitro studies have estimated extracellular Sap concentrations to reach approximately 800 ng/ml^41^, highlighting the relevance of the concentrations used in this study. Moreover, for phagocytic cells such as neutrophils, intracellular Sap6 concentrations may depend less on extracellular levels due to its accumulation in endosomes and lysosomes, where it can increase by as much as 4–9 times relative to its initial concentration in the surrounding solution.

This substantial intracellular accumulation likely enhances the immunomodulatory effects of Sap6, contributing to its ability to influence neutrophil function and alter the immune response during *C. albicans* infections.

Our research revealed that while some neutrophils release NETs in response to Sap6, a distinct subset undergoes apoptosis. These findings suggested that Sap6 exerts a pleiotropic effect depending on concentration, which influences the response mechanism of neutrophils. Both microscopic and cytometric analyses indicated that the activation of a specific response mechanism is closely linked with the presence and localization of the yeast protease either on the surface or inside the neutrophils. Cells exhibiting high activity of proapoptotic caspases 3/7, which undergo apoptosis, showed a marked increase in both surface and intracellular Sap6 localization over time.

Neutrophils are capable of actively removing foreign molecules through clathrin-dependent endocytosis^42^. Prebound molecules on the neutrophil surface are internalized into endosomes, which isolate and neutralize foreign compounds^43^. Our experiments, conducted in the continuous presence of extracellular Sap6, revealed active uptake of Sap6, indicating its accumulation in neutrophils. Approximately 30 min after contact, Sap6 was detectable within the neutrophils. Examination of the cell nucleus revealed that, in cells with Sap6 accumulation, neither a reduction in nuclear segmentation nor an increase in nuclear size was observed, which are typical features of NETosis resulting from chromatin decondensation. The morphological alterations noted in neutrophils, along with the lack of heightened SYTOX Green fluorescence, are consistent with the hallmarks of apoptosis, which include the formation of apoptotic bodies and gradual cellular breakdown, all of which occur while maintaining the fundamental architecture of the cell nucleus^44^.

The use of cytochalasin D, recognized as an inhibitor of endocytosis^42,45^, has confirmed that the increase in proapoptotic caspase activity is linked to the active internalization of Sap6 from the external environment. Actin reorganization is crucial for both phagocytosis and the multistep process of clathrin-dependent endocytosis^42^.

Cytometric and microscopic analyses revealed that Sap6 binding initially occurs on the surface of neutrophils, and this step is common and crucial for further NETosis^3^ as well as the redirection of neutrophils in the apoptosis pathway. We have shown that the interaction between Sap6 and the neutrophil surface that precedes endocytosis occurs with the participation of the Mac-1 receptor thus, the process of endocytosis is accelerated as a result of active endocytosis. Mac-1 is a multifunctional receptor that plays an important role in both NETosis and endocytosis^46^. Mac-1 receptor-mediated phagocytosis can induce neutrophil apoptosis^47^, and the expression of Mac-1 increases minutes after contact with a pathogenic agent, i.e., LPS^48^. Therefore, the regulatory mechanism of the neutrophil response involving Mac-1 is complex and is not completely understood. The differential roles of its subunits, CD11b and CD18, further highlight this complexity. CD11b, particularly its I-domain, is critical for ligand binding and subsequent internalization, as demonstrated by the complete inhibition of Sap6 binding following CD11b blockade with the M1/70 antibody^49–51^. In contrast, CD18 contributes to structural integrity and signaling, with evidence showing that its blockade using TS1/18 significantly inhibits adhesion and migration^52^ but has a less pronounced impact on Sap6 binding. Furthermore, CD11b plays a key role in the activation of NETosis by Sap6, while the contribution of CD18, although present, is relatively minor^3^. This indicates that CD11b is the primary mediator of Sap6 binding and internalization, with CD18 playing a supporting role in downstream signaling. These findings underline the dominant role of CD11b in Sap6-mediated responses and further emphasize the need for more detailed studies to elucidate the specific regulatory mechanisms governing Mac-1 function in neutrophil responses.

The observation that blocking CD11b does not completely abolish the observed effect suggests the involvement of additional receptors or alternative pathways in this process. Neutrophils demonstrate remarkable flexibility in their ability to capture extracellular proteins, which may explain the incomplete inhibition of internalization. One possibility is the contribution of other complement receptors, such as CR4 (CD11c/CD18), which is structurally similar to Mac-1 (CD11b/CD18) and recognizes overlapping ligands. Additionally, CR1 (CD35), which plays a key role in binding and opsonizing complement-coated particles, may contribute to the internalization process despite being less efficient than Mac-1. Together, these receptors might partially compensate for the reduced function of CD11b/CD18^53–56^.

Moreover, Fcγ receptors, including FcγRII (CD32) and FcγRIII (CD16), could play a significant role in the uptake of immune complexes or antibody-opsonized proteins. Numerous studies have shown that even when Mac-1 is blocked, Fcγ receptors can mediate internalization, ensuring continued functionality. Although their primary role is often initiating pro-inflammatory signaling, these receptors may facilitate some level of internalization under specific conditions^57,58^.

Finally, neutrophils may utilize alternative pathways such as micropinocytosis or clathrin-mediated endocytosis to compensate for receptor blockade. While these mechanisms are typically less efficient in neutrophils compared to macrophages or dendritic cells, they might still contribute to the observed effects^42^. Importantly, receptors on neutrophils often interact synergistically, enhancing internalization processes. This collaborative functionality may explain why partial inhibition of one pathway, such as CD11b/CD18 blockade, does not completely eliminate the ability of neutrophils to internalize proteins^59^. These observations highlight the importance of considering multiple pathways and mechanisms in the context of immune response studies.

Analysis of the colocalization of endosomes containing Sap6 and acidic granules suggested that fusion occurred, leading to the incorporation of biocidal compounds into the vesicles and altering the pH^60,61^. While acidification typically reduces the activity of inactivated compounds, the optimal pH for Sap6 activity is between 5 and 7^63,64^, indicating that these conditions may actually enhance their activity. This finding is in contrast to the results obtained for A549 epithelial cells, in which internalized Sap6 does not rapidly exit endosomes to induce apoptosis but rather accumulates within them^23^.

One of the strategies for combating pathogens and foreign molecules involves ROS generation by NADPH oxidase. Select oxidase subunits are located in the membrane of phagosomes and endosomes and form a fully functional complex upon neutrophil activation^64^. The release of ROS into intracellular vesicles minimizes potential damage to host tissues^31^. Additionally, oxidase plays a crucial role in the activation of NETosis, particularly through the ROS-dependent NETosis pathway^19^.

Our studies indicate that Sap6 triggers NADPH oxidase activation only within a specific concentration range of 1–10 ng/ml. At these levels, Sap6-induced ROS production leads to the activation of the ROS-dependent NETosis pathway, resulting in NET release^3^. However, higher concentrations of Sap6 fail to induce ROS and NET release, which is correlated with neutrophil apoptosis, but the underlying mechanism is unclear. Our detailed examination of ROS production revealed that increased extracellular concentrations of Sap6 significantly compromise the stability of NADPH oxidase. Chemical stimulation of neutrophils with phorbol esters (PMA), which act intracellularly by directly activating protein kinase C (PKC), leads to the formation of NADPH oxidase complexes and ROS production^65,66^. This approach allowed us to stimulate the NADPH oxidase system, bypassing transmembrane signaling pathways and surface receptors that Sap6 might degrade upon contact. The analysis of ROS production kinetics in neutrophils responding to PMA under varying concentrations of Sap6 revealed that the maximal response level (ROS production) decreased as the Sap6 concentration increased. These findings imply that Sap6 impacts the overall active NADPH oxidase pool but does not directly interfere with the enzymatic reaction; rather, it influences the ROS generation system, which results in the inhibition of NETosis.

To understand how Sap6 leads to the inhibition of ROS and NETosis and subsequently induces apoptosis, we utilized protease inhibition using pepstatin A. By eliminating the proteolytic properties of Sap6, we determined whether proteolysis or another protein interaction impedes the formation of a functional oxidase complex, thus blocking NADPH oxidase activity. These results confirmed that the proteolytic activity of Sap6 reduces ROS levels in neutrophils. When neutrophils were stimulated with PMA in the presence of inactivated Sap6, the amount of ROS released was comparable to that in the control group, which was activated without protease. Additionally, the inhibition of endocytosis showed that the proteolytic activity of Sap6 inside neutrophils inhibits ROS release. After cytochalasin D treatment, Sap6 did not significantly impact the production of NADPH oxidase-related ROS. Similarly, blocking the Mac-1 receptor prevents Sap6 from internalizing and hence from blocking ROS production mechanisms. These findings clarify that Sap6 modulates the neutrophil response by intracellularly inhibiting ROS generation through its proteolytic activity, paralleling other known intracellular microbial effects on neutrophil ROS systems^32^. Like Sap6 affects neutrophil function, other pathogens also modulate the ROS generation system in immune cells. For instance, *Anaplasma phagocytophilum* reduces CYBB (gp91^phox^) gene expression^67^. *Coxiella burnetii* impedes the translocation of p47^phox^ and p67^phox^ to endosomal membranes^68,69^. Additionally, the live vaccine strain of *Francisella tularensis* broadly inhibits respiratory burst activation by preventing the formation of flavocytochrome b_558_ and the recruitment of cytosolic p47^phox^ and p67^phox^ to phagosomes^70^. These examples highlight the diverse strategies pathogens employ to evade host immune responses.

Considering the intricate nature of the signaling pathways that trigger oxidative bursts and NETosis, we identified potential targets of the Sap6. Disruption of these targets could result in the suppression of ROS production and the release of NETs. We focused our analysis on common intracellular mediators involved in both processes.

Protein kinase C (PKC) plays a pivotal role in activating the ROS-dependent NETosis pathway by mobilizing NADPH oxidase. Our in vitro analysis of the potential degradation of PKC by Sap6 revealed that this mediator remains intact; it is neither degraded by the protease nor does its enzymatic activity change in the presence of Sap6. Additionally, spatial protection of PKC is evident, as it is localized outside of endosomes and remains separated from contact with Sap6. NADPH oxidase is partially exposed to the proteolytic activity of Sap6. This complex, comprising gp91^phox^, p22^phox^, p40^phox^, p47^phox^, and p67^[phox 71^, encounters Sap6 primarily at the endosome membrane. Endosomal conditions somewhat facilitate the proteolytic function of Sap6. Intracellular studies performed on neutrophils confirmed that both the gp91^phox^ and p67^phox^ subunits are inactivated directly in neutrophils as a result of the proteolytic activity of Sap6. Analysis of cell lysates collected after treatment with Sap6 revealed the formation of shorter polypeptide chains, indicating limited degradation of NADPH oxidase subunits. Additionally, in vitro experiments have shown that the p67^phox^ subunit is a specific target of the Sap6, leading to its fragmentation. An increased local concentration of Sap6 in endosomes facilitates the efficient degradation of NADPH oxidase, elucidating how Sap6 inhibits ROS production and consequently blocks NETosis. While similar actions by other microorganisms using proteolytic enzymes are not well documented, certain bacteria can enzymatically inhibit NADPH oxidase activity. For example, *Yersinia pestis* produces YopE, a protein with GTPase-like activity^72^, and Group A Streptococcus secretes streptolysin, a pore-forming toxin that inhibits NADPH oxidase activity through an unknown mechanism^73^. These defects in ROS production enable bacteria to persist, colonize various tissues, and potentially cause septicaemia^31^.

In our cellular assays, we demonstrated that proteolytically active Sap6 inside neutrophils impairs ERK1/2 kinase function. The activation of this kinase by PMA is significantly reduced in the presence of Sap6. Although ERK1/2 kinase activation can result from ROS production and is localized in the intracellular space away from endosomes, its decreased activity is likely a secondary effect of ROS inhibition. As a crucial apoptosis regulator, ERK1/2 might induce proapoptotic effects through indirect interactions with Sap6. This disruption of kinase function by Sap6 could lead to both the blockade of NETosis and the potential activation of apoptosis in neutrophils^28,74^.

To determine the alternative effects of Sap6 on neutrophils, we specifically analyzed the direct effect of Sap6 on NETosis. Our findings revealed that the extent of PMA-induced NETosis in the presence of Sap6 is contingent on the concentration. Additionally, the quantity of NETs released was found to be proportional to the level of ROS production. These findings support the conclusion that NETosis inhibition is a secondary consequence of NADPH oxidase impairment. By supplementing extracellular ROS with hydrogen peroxide, even in the presence of Sap6, neutrophils were able to release NETs, indicating that ROS deficiency is the limiting factor in the mechanism of NETosis. ROS supplementation restored full functionality, suggesting that Sap6, in addition to NADPH oxidase, does not damage other components of the NETosis signaling pathway. These findings mirror observations in chronic granulomatous disease (CGD), where H_2_O_2_ restored NETosis in neutrophils from affected patients^75^. Notably, delayed H_2_O_2_ application does not reestablish NET release, likely due to proapoptotic caspase activation within that hour, which effectively inhibits NETosis.

In summary, *C. albicans* utilizes Sap6 as a defensive mechanism against the host immune system. Concurrently, with the growth of the *C. albicans* biofilm, an increased amount of Sap6 was observed in the extracellular space^76^. Neutrophils, as phagocytes, recognize the RGD fragment of the pathogenic protein and internalize it through the Mac-1 receptor. This process results in protein localization within endosomes, leading to the aggregation of Sap6. Pathogen contact triggers the NETosis mechanism, which includes the activity of NADPH oxidase within endosomes. Initially, low extracellular Sap6 concentrations allow NETosis to proceed. However, an increase in Sap6 concentration leads to the degradation of NADPH oxidase subunits, disrupting the ROS-dependent NETosis pathway. Consequently, NET release is inhibited, and proapoptotic caspases are activated. This ‘Trojan horse’ mechanism illustrates the delicate balance in the immune response against pathogens such as *C. albicans*, where actions intended to counteract pathogens can become counterproductive, blurring the line between effective neutrophil action and complete neutrophil inactivation.

## Materials and methods

### Neutrophil isolation

Human polymorphonuclear cells (PMNs) were isolated from EDTA-treated whole-blood samples obtained from healthy donors via the Regional Blood Donation Center (Krakow, Poland). Whole blood (20 ml) was collected into 50 ml tubes containing EDTA at a final concentration of 5 mM and maintained at room temperature throughout the isolation procedure. The tubes were centrifuged at 300 × g for 15 min, the plasma layer was removed, and the bottom layer containing leukocytes and erythrocytes was restored to the original volume of 20 ml with phosphate-buffered saline (PBS) (Sigma‒Aldrich, St. Louis, MO) and gently mixed by inverting the tube four times. The cell suspension was carefully layered on 10 ml of Lymphocyte Separation Medium 1077 (Sigma‒Aldrich) and centrifuged at 300 × g for 30 min. The supernatant was removed, and 20 ml of 1% polyvinyl alcohol (Sigma‒Aldrich) was gently mixed with the suspension by inverting the mixture four times. The tubes were allowed to stand for 20 min at room temperature to enable erythrocyte sedimentation. The supernatant containing the PMNs was then collected into a fresh tube and centrifuged at 110 × g for 5 min. The supernatant was subsequently removed, and the erythrocytes in the cell pellet were lysed by the addition of 1 ml of red blood lysis buffer (Roche Diagnostics, Mannheim, Germany) for 10 min. Nine milliliters of PBS was added to stop the lysis reaction, and the mixture was centrifuged at 110 × g for 5 min. The supernatant was removed, and the cell pellet was resuspended in 1 ml of RPMI-1640 medium without phenol red (Biowest, Nuaille, France). Neutrophil purity was routinely assessed by forward- and side-scatter flow cytometric analyses. This neutrophil preparation method yields a > 95% pure population of cells.

### Production and isolation of recombinant Sap6

The Sap6 enzyme was obtained according to the method described previously^8,77^ after its overexpression in the *Pichia pastoris* system (Invitrogen, Waltham, MA). The homogeneity of the purified proteins was checked by sodium dodecyl sulfate‒polyacrylamide gel electrophoresis (SDS‒PAGE), and their proteolytic activities were assayed on the boron–dipyrromethene FL casein substrate (Invitrogen) in 0.1 M buffer at pH 5.0^8^.

### Fluorescent labeling of Sap6

For protein labeling, Sap6 was predialyzed against 0.1 M bicarbonate buffer (pH 8.3). Then, a solution of NHS-fluorescein (FITC, Thermo Fisher Scientific) or Alexa Fluor 555 NHS Ester (Thermo Fisher Scientific) prepared in dimethyl sulfoxide (1 mg/100 µL) was added, maintaining a proper ratio. The reaction mixture was incubated for 4 h at 4 °C or 1 h at 37 °C for NHS-fluorescein or NHS-Alexa Fluor 555, respectively. The labeled proteins were then dialyzed against PBS (pH 7.4) at 4 °C for 48 h.

### Caspase 3/7 activity assay

Neutrophils used for apoptosis analysis were prepared according to the planned protocol.

#### Time-dependent analysis

Neutrophils (1 × 10^6^ cells/sample) were resuspended in 50 µl of RPMI-1640 medium containing CellEvent™ Caspase-3/7 Green Detection Reagent (Sigma‒Aldrich) at a concentration of 2 µM in Eppendorf tubes and incubated for 30 min at 37 °C with 5% CO_2_. Then, Sap6 at a concentration of 200 ng/ml was added to each tube in a volume of 50 µl (with a final concentration of 100 ng/ml), and the mixture was incubated at 37 °C with 5% CO_2_. Every 15 min, subsequent samples were analyzed via flow cytometry (LSR Fortressa, BD, San Jose, CA, USA).

#### The effect of endocytosis analysis

One part of the neutrophils was incubated for 15 min with the endocytosis inhibitor cytochalasin D (cytD) at a concentration of 5 µM, after which the cells were washed with PBS and stained with CellEvent™ Caspase-3/7 Green Detection Reagent. The subsequent procedure was analogous to that described above, except that all the samples were incubated for 1 h before fixation.

### Internalization of Sap6

#### Microscope analysis

Neutrophils (2 × 10^5^ cells/well) were resuspended in 50 µl of RPMI-1640 medium and placed in the wells of a 96-well glass-bottom microplate (CellVis, Mountain View, CA, USA), after which fluorescently labeled Sap6 at a final concentration of 100 ng/ml was added. The cells were imaged during incubation for 45 min using an Olympus IX73 fluorescence microscope, and images were taken every 6 min. The background signal threshold for the Alexa 555 fluorescence channel was adjusted to exclude signals from the free Sap6 in the surrounding solution. This setup allowed for the detection of changes in the localized concentration of Sap6, such as accumulation or surface binding, as indicated by an increase in fluorescence intensity.

#### Flow cytometry analysis

##### Time-dependent analysis

Neutrophils (1 × 10^6^ cells/sample) were resuspended in 50 µl of RPMI-1640 medium containing Sap6 at a concentration of 100 ng/ml in Eppendorf tubes. The samples were incubated at 37 °C with 5% CO_2_. Every 15 min, 50 µl of 7.4% paraformaldehyde (3.7% final concentration) was added to the subsequent samples to fix the cells. At the end of the incubation, the cells were washed 3 times with PBS, centrifuged at 300 × g for 5 min each, resuspended in 500 µl of PBS and analyzed cytometrically.

##### Concentration-dependent analysis

One part of the neutrophils was incubated for 15 min with the endocytosis inhibitor cytochalasin D (cytD) at a concentration of 5 µM, after which the cells were washed with PBS. Control and CytD-treated neutrophils were resuspended in 50 µl of RPMI-1640 medium (1 × 10^6^ cells/sample) containing Sap6 at concentrations ranging from 0 to 600 ng/ml and incubated for 1 h at 37 °C with 5% CO_2_. The samples were subsequently treated and analyzed as described above.

### Identification of the neutrophil receptors involved in Sap6 binding

To identify neutrophil surface receptors involved in Sap6 binding, cells were preincubated with antibodies blocking selected receptors before contact with the Sap6. Neutrophils (1 × 10^6^) were preincubated for 30 min at 37 °C in RPMI-1640 medium supplemented with 1 µg/ml blocking antibodies against TLR2, TLR4 (InvivoGen, Toulouse, France), CD11b, CD16, CD18 (BioLegend, San Diego, CA, USA) or the isotype control antibody IgG (Abcam, Cambridge, UK). After incubation, the cells were washed and resuspended in 50 µl of 100 ng/ml Sap6 solution in RPMI-1640 and incubated for 30 min. After incubation, the neutrophils were washed and analyzed cytometrically as described above.

### Colocalization of Sap6 and endosomes

Colocalization analysis of the Sap6 and acidic endosomes inside neutrophils was performed using fluorescence microscopy. For this purpose, a portion of the neutrophils was incubated for 15 min with LysoTracker Yellow tracer (Sigma‒Aldrich) according to the manufacturer’s instructions. Then, 2 × 10^5^ cells were transferred to the wells of a 96-well glass-bottomed black plate (CellVis) in a volume of 50 µl. Fluorescently labeled (FITC) Sap6 at a final concentration of 100 ng/ml was added to the selected wells and incubated for 30 min at 37 °C and 5% CO2. The samples were then washed 3 times and fixed with 3.6% paraformaldehyde for 10 min, after which the cells were removed, and a few drops of Prolog Gold Antifade Mountant (Invitrogen) were added to the wells. Slides were prepared in this way and analyzed using an Olympus IX73 fluorescence microscope and CellSens software. The obtained images were deconvoluted using the implemented software.

### ROS production

#### Chemiluminescence measurements

Neutrophils (2 × 10^5^ cells/well) preincubated with Sap6 as described above were suspended in 160 µl of Krebs–Ringer phosphate buffer containing freshly prepared luminol solution (10^–6^ M) and allowed to settle for 15 min at 37 °C and 5% CO2 in the wells of a 96-well white microplate, after which 25 nM PMA was added. Untreated neutrophils were used as a negative control, but stimulation with 25 nM PMA without Sap6 was used as a positive control. The chemiluminescence of the luminol was recorded for one hour with a one-second integration time using a BioTek Synergy H1 microplate reader.

#### Fluorescence measurements

ROS production was detected via a fluorescence assay using rhodamine-123 (Invitrogen, Waltham, MA) to label neutrophils. The cells were incubated with the indicator at a final concentration of 5 µM for 15 min at 37 °C. The cells were then washed with PBS and treated as described above, depending on the experiment, i.e., with an endocytosis inhibitor or a CD11b receptor-blocking antibody and incubated with Sap6 at selected concentrations. After the experiment, the neutrophils were fixed with 3.6% paraformaldehyde and analyzed via flow cytometry.

### PKC activity studies

The impact of Sap6 on PKC was monitored using PepTag® Non-Radioactive Protein Kinase Assays (Promega, Madison, WI, USA). Protein kinase C (2.5 µg/ml) was incubated with Sap6 at 100 ng/ml and 300 ng/ml in acetate buffer solution (pH 5.5) for 30 min at 37 °C. After incubation, pepstatin A at a final concentration of 10 µM was added to the samples to block further Sap6 activity. Next, 4 µL of the prepared sample (or protein kinase C at a concentration of 2.5 µg/mL, as a positive control) was added to the reaction mixture containing 5 µL of PepTag® PKC Reaction Buffer, 2 µg of PepTag® C1 peptide and 5 µL of sonicated PKC activator, followed by incubation at 30 °C for 30 min. Then, the reaction was stopped by placing the tube in a 95 °C heating block for 10 min. One microliter of 80% glycerol was added to each sample, and the samples were electrophoretically separated on a 0.8% agarose gel at 100 V for 15 min. The phosphorylated peptide migrated to the cathode (+), while the nonphosphorylated peptide migrated to the anode (–). The gel was photographed on a transilluminator.

## ERK1/2 activity studies

The amount of total and phosphorylated ERK1/2 was quantified using a SimpleStep ELISA Kit (Abcam). Neutrophils (1 × 10^6^/well in 200 µL of RPMI-1640) with active or blocked endocytosis (as described previously) were incubated with active or inhibited Sap6 at a concentration of 1200 ng/ml in a 24-well microplate for 30 min at 37 °C in 5% CO2. Then, the neutrophils were chemically activated with 25 nM PMA, and after 1 h of incubation under the conditions above, the cells were lysed using cell extraction buffer (Abcam). The protein concentration in the lysate was determined using the Bradford assay^78^. Then, 50 µL of lysate was mixed with 50 µL of antibody cocktail (anti-ERK1/2– total or anti-pT202/Y204–phosphorylated ERK1/2 provided by the manufacturer) in the wells of a SimpleStep precoated 96-well microplate according to the manufacturer’s instructions. After 1 h of incubation with gentle shaking at room temperature, the wells were washed 3 times with PBS, after which TMB (3,3′,5,5′-tetramethylbenzidine) was added. After 15 min, the reaction was stopped with a Stop solution, and the absorbance at 450 nm was recorded using a BioTek Synergy H1 microplate reader.

### NADPH degradation assay

#### Protein assay

p67^phox^ (1 µg) (Abcam) was incubated with Sap6 at a substrate: enzyme molar ratio of 50:1 in 50 mM sodium phosphate buffer (pH 7.0) at 37 °C for 3 h. p67^phox^ degradation products were separated by SDS‒PAGE under reducing conditions in the Laemmli system using a 12% separating gel and then visualized via silver staining.

For the sample containing the proteinase inhibitor, Sap6 was first preincubated for 30 min on ice with 10 µM pepstatin A, followed by p67^phox^ degradation was analyzed as described above.

#### Cell assay

Neutrophils (2 × 10^5^ cells/sample) were incubated with Sap6 at a concentration of 1200 ng/ml for 3 h, as described above. The cells were then centrifuged, resuspended in RIPA lysis buffer (100 mM Tris-HCl, 150 mM NaCl, 1% deoxycholic acid, 1% Triton X-100, 0.1% SDS, 10 µM pepstatin A and protease and phosphatase inhibitors) and sonicated five times for 5 seconds each on ice. The lysates were centrifuged (13,000 × g, 15 min), and the supernatant was gently transferred to new tubes. The supernatant proteins were separated via SDS‒PAGE under reducing conditions in a Laemmli system via a 12% separating gel and subsequently transferred to nitrocellulose membranes. The membranes were blocked in TBST (25 mM Tris, pH 7.2; 150 mM NaCl; 0.1% Tween) with 3% nonfat dry milk for 1 h at room temperature and incubated overnight at 4 °C in blocking buffer with an anti-p67^phox^ antibody (R&D Systems, Minneapolis, MN, USA) (1:400) or an anti-gp91^phox^ antibody (Abcam) (1:1000). Then, the membranes were washed and incubated with donkey anti-sheep (R&D Systems) or goat anti-rabbit (Thermo Fisher) HRP-conjugated antibodies. A chemiluminescence substrate (Cyanagen, Bologna, Italy) was used to detect HRP. The relative intensities of the immunoreactive bands were quantified by scanning densitometry using NIH ImageJ v.1.53i analysis software.

### NETosis analysis assay

Neutrophils (2 × 10^5^ cells/well) were resuspended in 50 µl of RPMI-1640 medium and placed in the wells of a 96-well glass bottom microplate (CellVis).

Depending on the experiment, Sap6 was added to the wells at concentrations ranging from 12 to 1200 ng/ml or at a fixed concentration of 1200 ng/ml, and the plates were incubated for 30 min at 37 °C with 5% CO_2_. Then, 50 µl of PMA at a final concentration of 25 nM was added to the wells. In one experiment, 10 µl of H_2_O_2_ (0.03% final concentration) was added simultaneously with PMA or after 1 h. After another 3 h of incubation, the wells were gently washed with PBS, 50 µL of micococcal nuclease (MNase, 1 U/mL) was added to cleave and release small fragments of extracellular DNA, and the microplates were incubated at 37 °C for 20 min. The enzymatic reaction was stopped by adding EDTA solution (100 µg/mL), and after centrifugation (350× g, 5 min), 50 µL of the supernatant was transferred to a 96-well black microplate containing Sytox Green at a final concentration of 1 µM. Fluorescence was measured using a Biotek Synergy H1 microplate reader at an excitation wavelength of 465 nm and an emission wavelength of 525 nm.

### Simultaneous microscopic analysis of apoptosis, NETosis, and Sap6 internalization

Cell apoptosis was analyzed by measuring proapoptotic caspase 3/7 activity based on fluorescence, while NET release was observed by DNA labeling with DAPI. For microscopic analyses, neutrophils (2 × 10^5^ cells/well) were resuspended in 50 µl of RPMI-1640 medium containing CellEvent™ Caspase-3/7 Green Detection Reagent (Sigma‒Aldrich) at a concentration of 2 µM, placed in the wells of a 96-well glass-bottom microplate (CellVis) and incubated for 30 min at 37 °C with 5% CO_2_. Labeled Sap6 at 100 ng/ml and DAPI at a final concentration of 0.1 µg/ml (Sigma‒Aldrich) were subsequently added to the wells, which were subsequently imaged for 45 min using an Olympus IX73 fluorescence microscope, after which images were taken every 6 min.

### Analysis of *C. albicans* growth

The growth of C. albicans was analyzed using a co-incubation model with neutrophils. Briefly, C. albicans cells (1 × 10^7^) were co-incubated with neutrophils at a multiplicity of infection (MOI) of 1 in 96-well plates containing RPMI-1640 medium. The plates were maintained at 37 °C in a microplate reader equipped with an environmental control system to stabilize temperature and atmospheric conditions. Optical density (OD) at 600 nm was measured hourly over a 19-hour incubation period to monitor fungal growth. Negative controls consisted of wells containing neutrophils alone, while positive controls contained C. albicans without neutrophils. Experimental wells included *C. albicans* co-incubated with neutrophils, as well as C. albicans co-incubated with neutrophils pre-incubated with Sap6 (120 ng/ml) for 30 min prior to the experiment. These conditions allowed for the assessment of the impact of neutrophils and Sap6 on fungal growth dynamics.

### Statistical methods and graphics

Each of the experiments was repeated at least three times, each yielding consistent results. Two replicates were performed for each experiment.

All the statistical analyses were performed with GraphPad Prism 8 software (GraphPad Software, CA, USA). The statistical significance was assessed by ANOVA and Dunnett’s multiple comparisons post hoc test.

The figures were prepared in GraphPad Prism.

## Electronic supplementary material

Below is the link to the electronic supplementary material.

Supplementary Material 1

## Data Availability

The datasets used and/or analysed during the current study available from the corresponding author on reasonable request.
