# Supplementary material for: Candida albicans aspartyl protease (Sap6) inhibits neutrophil function via a “Trojan horse” mechanism
Source: Sci Rep. 2025 Feb 26;15:6946. doi: 10.1038/s41598-025-91425-x (PMC11865311; doi:10.1038/s41598-025-91425-x)
Supplement: Supplementary file 1 — Supplementary Material 1 [file 41598_2025_91425_MOESM1_ESM.docx]

Supp, fig. 1

**
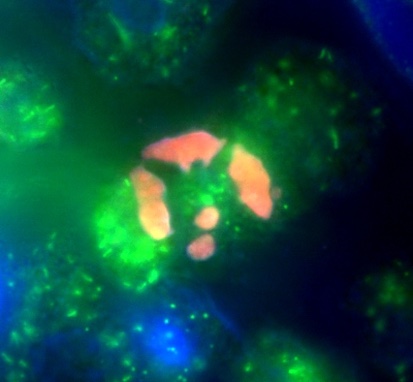

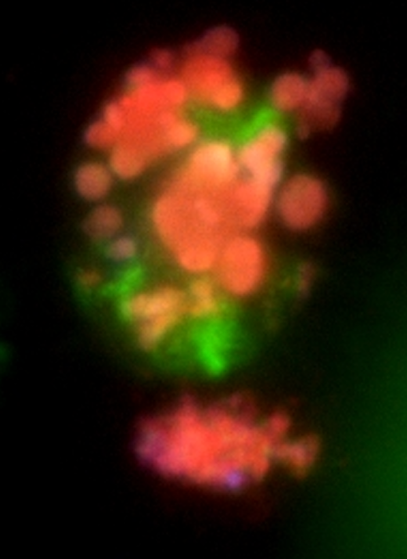
**

**Apoptosis of neutrophils in contact with Sap6.** Neutrophils were stained with CellEvent^TM^ Caspase-3/7 Green Detection Reagent (green channel) and incubated for 1 h with fluorescently labeled Sap6 at a concentration of 100 ng/ml (red channel). DAPI dye (blue) was then added, and the slides were imaged using fluorescence microscopy.

Supp, mov. 1


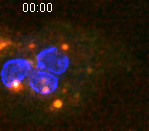


**Movie 1. Visualization of neutrophil responses to Sap6.** Apoptosis was indicated by chromatin condensation (blue, DAPI) and activation of caspase 3/7 (green, Sytox Green). The presence of Sap6 is shown in red (Alexa Fluor 647).

Supp. Mov. 2


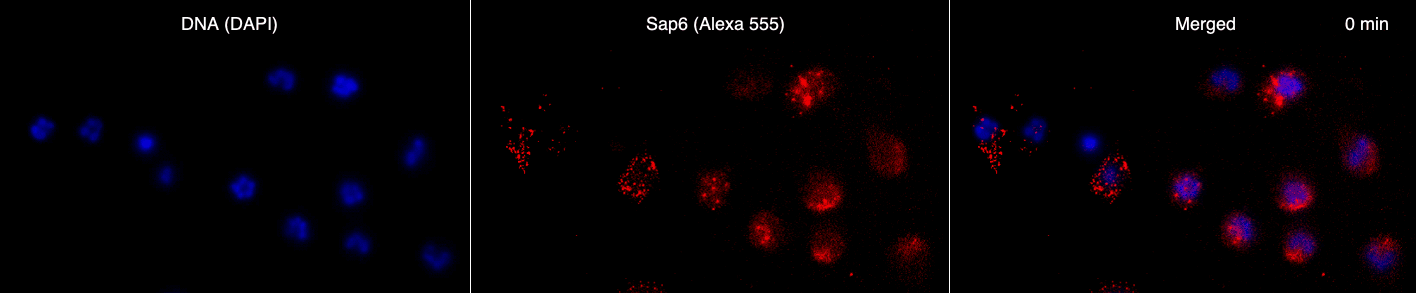


**Movie 2. Internalization of Sap6 by neutrophils.** Neutrophils labeled with DAPI (blue) were incubated with fluorescently labeled Sap6 (red) at a concentration of 100 ng/ml for 45 minutes. Subsequent microscopy images were taken every 6 minutes with the same microscope and camera settings. The video shows the process of protein accumulation inside neutrophils. The intensity of fluorescence is influenced by the concentration of the fluorophore, instrumental settings, and molecular factors. During the measurement, both the microscope and detector settings were kept constant, and the protein containing the fluorophore was continuously present in the solution. Therefore, the appearance and subsequent increase in the brightness of these dots during the observation period are indicative of a localized increase in the concentration of labeled proteins.

Supp. fig. 2

p67^phox^


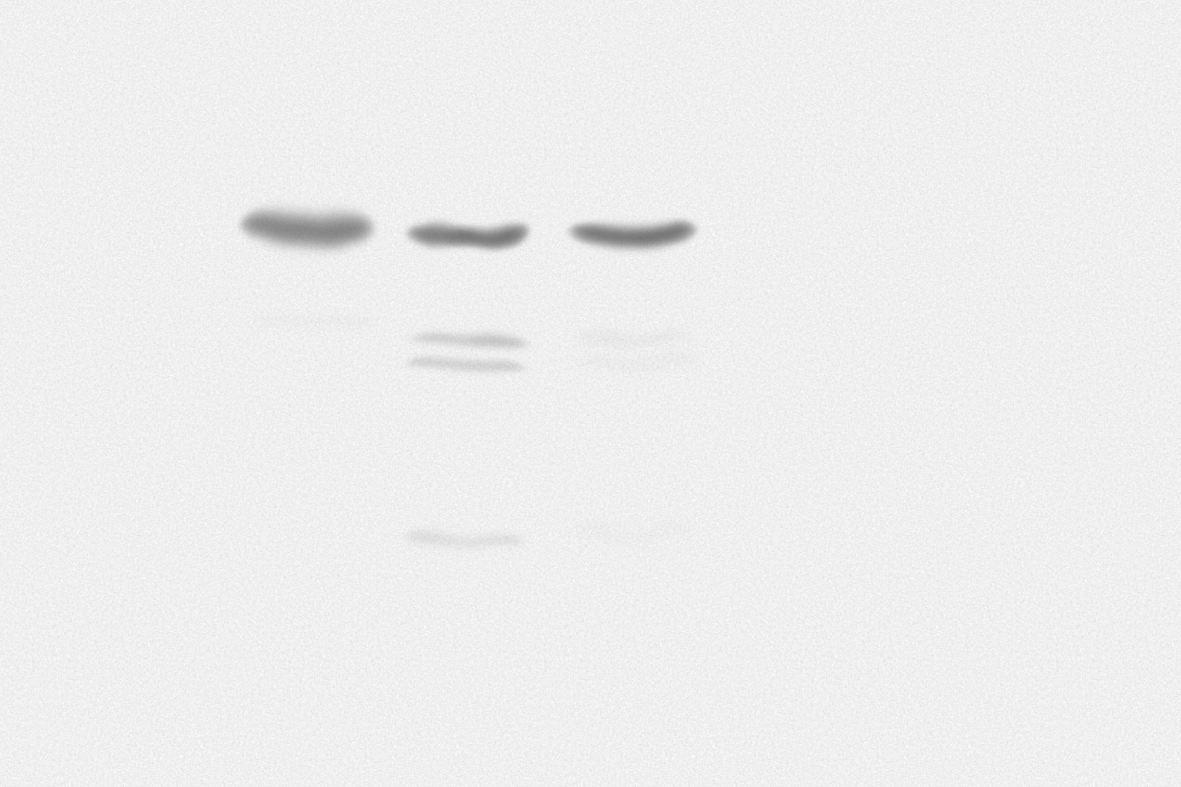


gp91^phox^


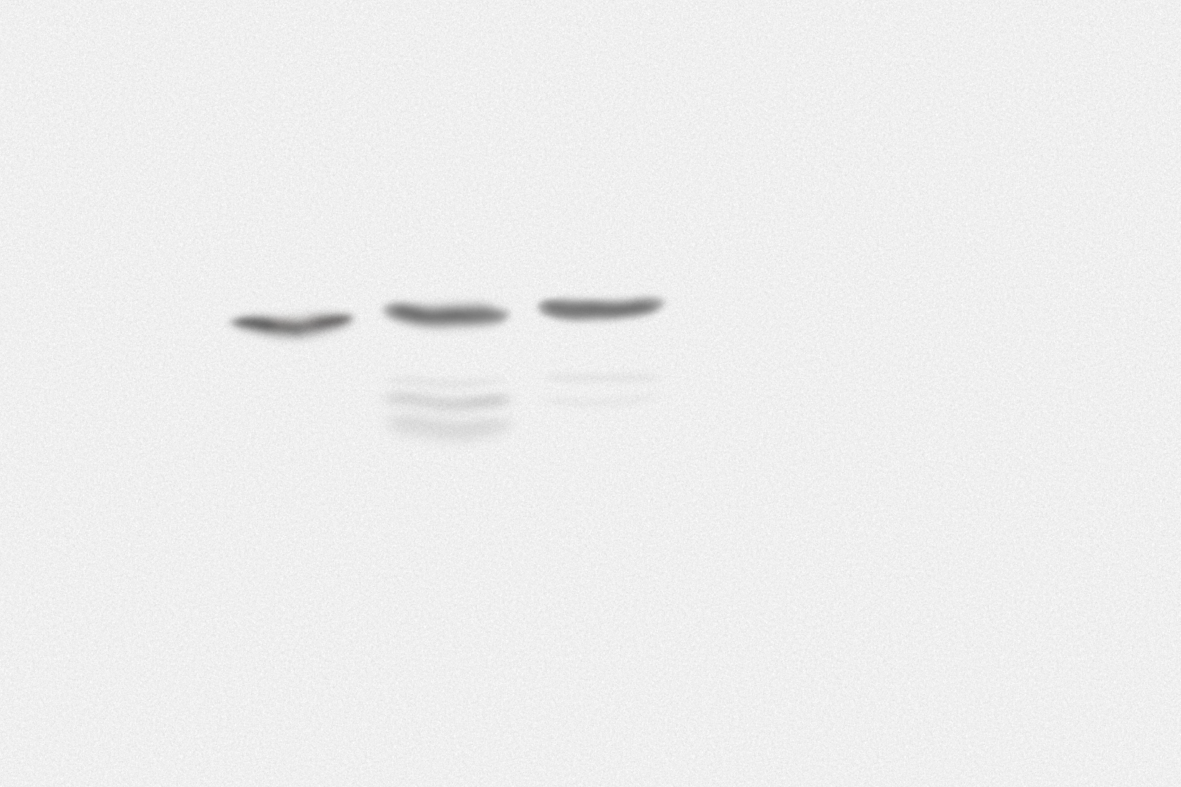


**Full size images of western blott, figure 7 from manuscript.**

Supp. fig. 3


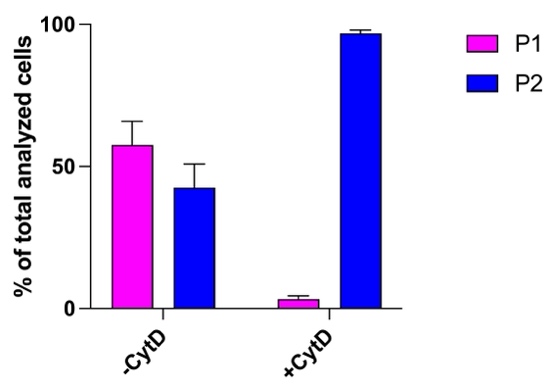


**Distribution of P1 and P2 cell populations.** The bar chart illustrates the percentages of P1 (pink) and P2 (blue) fractions among the total analyzed cells, with and without Cytochalasin D (CytD) treatment. Data are expressed as mean ± standard error of the mean (SEM).

Supp. fig. 4


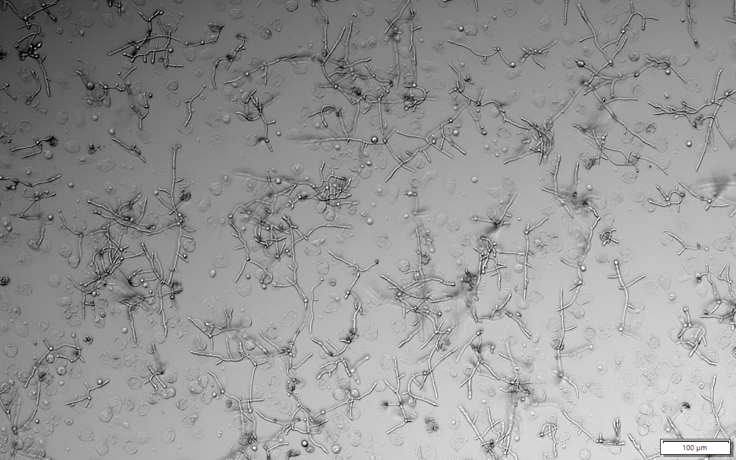


3h


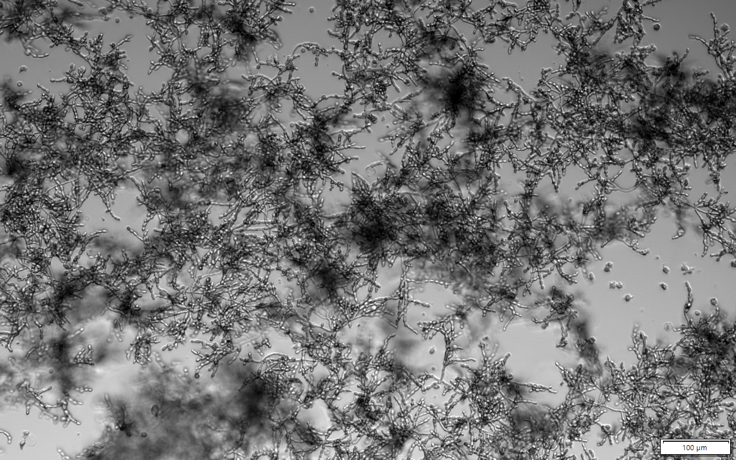


19h

**Morphological changes of *C. albicans* during incubation with neutrophils.** Representative images of *C. albicans* after 3 and 19 hours of incubation with neutrophils at 37°C in RPMI-1640 medium. The images illustrate the morphological changes of *C. albicans* over time in the presence of neutrophils.

Supp. fig. 5


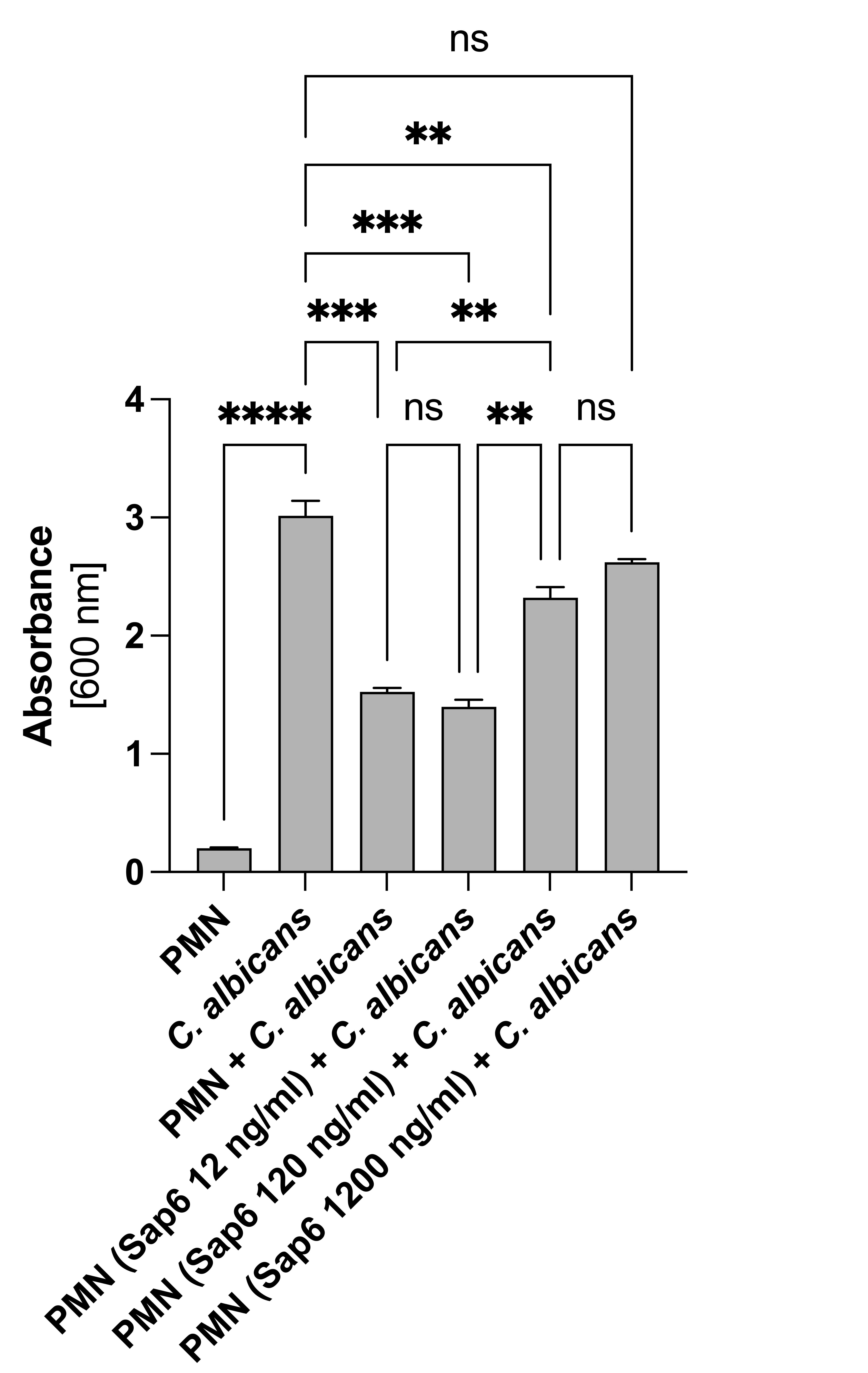


**Effect of Sap6 concentrations on *C. albicans* growth after co-incubation with neutrophils.** Growth of *C. albicans* co-incubated with neutrophils pretreated with different concentrations of Sap6, measured after 8 hours of incubation. The bar graph illustrates the effects of varying Sap6 concentrations on neutrophil-mediated fungal growth inhibition, highlighting the dose-dependent effects of Sap6 on neutrophil-mediated fungal inhibition. Data indicate that higher concentrations of Sap6 modulate neutrophil activity, influencing fungal growth dynamics. Data are presented as mean ± standard error of the mean (SEM).

Supp. fig. 6


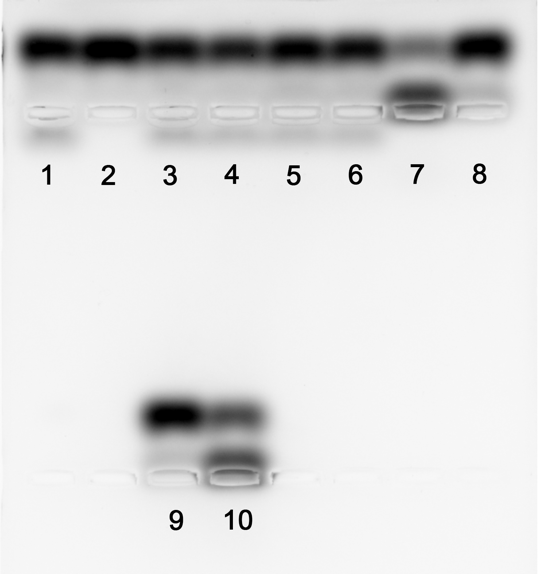


**Full size image of electrophoresis, figure 6A from manuscript.** Lane descriptions:

1. PKC + C1 peptide (positive control)
2. C1 peptide (negative control)
3. PKC + Sap6 (100 ng/ml) – incubation for 30 minutes, followed by the addition of the pepA inhibitor (10 µM) + C1 peptide
4. PKC + Sap6 (300 ng/ml) – incubation for 30 minutes, followed by the addition of the pepA inhibitor (10 µM) + C1 peptide
5. Sap6 (100 ng/ml) + pepA (10 µM) – preincubation for 15 minutes, followed by the addition of PKC – incubation for 30 minutes, then addition of C1 peptide
6. Sap6 (300 ng/ml) + pepA (10 µM) – preincubation for 15 minutes, followed by the addition of PKC – incubation for 30 minutes, then addition of C1 peptide
7. Sap6 (100 ng/ml) + C1 peptide (no pepA
8. Sap6 (100 ng/ml) + pepA (10 µM) – preincubation for 15 minutes, then addition of C1 peptide
9. Sap6 (300 ng/ml) + pepA (10 µM) – preincubation for 15 minutes, then addition of C1 peptide
10. Sap6 (300 ng/ml) + C1 peptide
